# Individual-level risk factors for suicide mortality in the general population: an umbrella review

**DOI:** 10.1016/S2468-2667(23)00207-4

**Published:** 2023-10-26

**Authors:** Louis Favril, Rongqin Yu, John R Geddes, Seena Fazel

**Affiliations:** a Institute for International Research on Criminal Policy, Faculty of Law and Criminology, Ghent University, Ghent, Belgium; b Department of Psychiatry, University of Oxford, Oxford, UK

## Abstract

**Background:**

Deaths by suicide remain a major public health challenge worldwide. Identifying and targeting risk factors for suicide mortality is a potential approach to prevention. We aimed to summarise current knowledge on the range and magnitude of individual-level risk factors for suicide mortality in the general population and evaluate the quality of the evidence.

**Methods:**

In this umbrella review, five bibliographic databases were systematically searched for articles published from database inception to Aug 31, 2022. We included meta-analyses of observational studies on individual-level risk factors for suicide mortality in the general population. Biological, genetic, perinatal, and ecological risk factors were beyond the scope of this study. Effect sizes were synthesised and compared across domains. To test robustness and consistency of the findings, evidence for small-study effects and excess significance bias (ie, the ratio between the overall meta-analysis effect size and that of its largest included study) was examined, and prediction intervals were calculated. Risk of bias was assessed by the Risk of Bias in Systematic Reviews instrument. The protocol was pre-registered with PROSPERO (CRD42021230119).

**Findings:**

We identified 33 meta-analyses on 38 risk factors for suicide mortality in the general population. 422 (93%) of the 454 primary studies included in the meta-analyses were from high-income countries. A previous suicide attempt and suicidal ideation emerged as strong risk factors (with effect sizes ranging from 6 to 16). Psychiatric disorders were associated with a greatly elevated risk of suicide mortality, with risk ratios in the range of 4–13. Suicide risk for physical illnesses (such as cancer and epilepsy) and sociodemographic factors (including unemployment and low education) were typically increased two-fold. Contact with the criminal justice system, state care in childhood, access to firearms, and parental death by suicide also increased the risk of suicide mortality. Among risk factors for which sex-stratified analyses were available, associations were generally similar for males and females. However, the quality of the evidence was limited by excess significance and high heterogeneity, and prediction intervals suggested poor replicability for almost two-thirds of identified risk factors.

**Interpretation:**

A wide range of risk factors were identified across various domains, which underscores suicide mortality as a multifactorial phenomenon. Prevention strategies that span individual and population approaches should account for the identified factors and their relative strengths. Despite the large number of risk factors investigated, few associations were supported by robust evidence. Evidence of causal inference will need to be tested in high-quality study designs.

**Funding:**

Wellcome Trust.

## Introduction

Death by suicide remains a global public health concern.^1^ The causes of suicide mortality are complex, involving a wide range of factors across biological, clinical, psychosocial, and environmental domains.^2^, ^3^, ^4^ One key approach to inform suicide prevention efforts includes the identification of risk factors, which can help determine the nature and type of interventions required.^5^ The number of primary studies examining risk factors for suicide mortality has increased markedly in recent decades,^6^ many of which have been quantitatively summarised in meta-analyses. Previous attempts to review this meta-analytic literature have focused on specific factors, including psychiatric disorders^7^ and cancer.^8^ However, there is a need to assess the full range of risk factors examined in meta-analyses to allow for a comparison of their relative importance. We have therefore conducted an umbrella review of meta-analyses to synthesise the state of knowledge on individual-level risk factors for suicide mortality and evaluate the quality of the underlying review evidence. Findings could inform how public health and clinical services prioritise interventions based on comparative risks, help policy makers to target resources most effectively, and draw attention to gaps in the research literature.

## Methods

We conducted an exposure-wide umbrella review^9^ to systematically collect and review published meta-analyses examining risk factors for suicide mortality. The study protocol was pre-registered on PROSPERO (CRD42021230119). The PRISMA guidelines^10^ were followed (appendix pp 2–4).

**Research in context**

**Evidence before this study**

We searched PubMed, Web of Science, Embase, Global Health, and PsycINFO for umbrella reviews, published from database inception to March 1, 2023, without language limitations, using the search terms (suicid*) AND (umbrella OR meta*). Our search identified two umbrella reviews that synthesised evidence on selected risk factors for suicide mortality (psychiatric disorders and cancer). These reviews did not assess the robustness and consistency of the evidence. We did not identify any umbrella review that captured the wide range of factors associated with suicide mortality.

**Added value of this study**

In this umbrella review of 33 meta-analyses synthesising research over five decades, we have presented an overview of 38 individual-level risk factors for suicide mortality and appraised the quality of the evidence base. We identified associations with a broad range of health conditions and comorbidities, strongest for psychiatric ones, and weaker effects for sociodemographic factors. However, the quality of the research was limited by excess significance and high heterogeneity between studies, and prediction intervals suggested poor replicability.

**Implications of all the available evidence**

The available evidence underscores suicide mortality as a multifactorial phenomenon, involving a wide range of risk factors across various domains. Individual and population-based strategies need to consider modifiable factors with the strongest links to and population impact on suicide mortality. Given the major burden of suicide mortality on public health, higher quality research (including in low-income and middle-income countries) is needed to inform prevention and intervention.

### Search strategy and selection criteria

We did a keyword search of titles and abstracts in five electronic databases (Web of Science, PubMed, PsycINFO, Embase, and Global Health) for meta-analyses published from database inception to Aug 31, 2022. The same search string was used for each database: (suicid*) AND (meta-analy* OR meta-rev* OR meta-reg* OR meta-syn*). We used forward and backward citation chaining to supplement our search, and reference lists of relevant reviews were manually searched. Targeted searches were also conducted to identify specific risk factors not identified in the main search.

Eligible studies were systematic reviews with meta-analysis of observational studies that examined individual-level risk factors for suicide mortality in the general population and provided a pooled effect size for the association between a risk factor and suicide mortality. Systematic reviews without meta-analysis were excluded as we intended to provide quantitative comparisons and test quality. We did not consider meta-analyses that only reported on prevalence or incidence rates (rather than associations). Eligibility was assessed by LF and discussed with SF.

Suicide mortality was the outcome of interest. Meta-analyses of risk factors for suicide attempt or self-harm were not included because associations have been shown to differ by outcome.^6^ Clear examples include age and sex, for which associations are different for suicide mortality compared with non-fatal suicidal behaviour.^2^ When meta-analyses examined the association between a given risk factor and multiple suicide-related outcomes (commonly ideation, attempt, and death), we only included the data specifically relating to suicide mortality (although in a secondary analysis, we compared effect sizes for these different outcomes when reported in the same meta-analysis). Reviews that solely focused on suicide deaths by a specific method (eg, hanging, poisoning, or firearms) were excluded because these are differentially associated with specific demographic and clinical factors,^11^, ^12^ which would limit generalisability (eg, compared with other methods, people who die by firearms are less likely to have a history of suicide attempts or mental illness).

The focus was on the general population, according to three inclusion criteria. First, meta-analyses were eligible if included samples were mainly, or solely, drawn from the general population, as opposed to clinical (eg, psychiatric patients^13^) or other high-risk populations (eg, prisoners^14^ and people who self-harmed^15^). These are selected populations with background risk factors that are different to the general population, so associations will probably be restricted to particular factors that are unique to these populations (eg, violent offending in prisoners) or dilute effects when the underlying prevalence is different (eg, mood disorders in people who have self-harmed). Meta-analyses that primarily included clinical and high-risk samples were only retained if they reported a separate estimate for the general population in subgroup analyses. We excluded the 2017 meta-analysis by Franklin and colleagues^6^ and their related publications as they included, for the most part, clinical and high-risk samples and did not provide subgroup analyses for suicide mortality in general population samples (appendix pp 5–8). Second, we excluded meta-analyses exclusively focusing on specific age groups (eg, adolescents^16^ or middle-aged adults^17^), as risk factors are known to vary by age.^3^ Third, meta-analyses examining risk factors for deaths by suicide limited to a single country or a specific geographical region (eg, North America) were not considered because of threats to generalisability given the considerable cross-national variation in suicide rates.^1^ In addition, risk factors with data obtained from fewer than three primary studies were excluded. For instance, a meta-analysis on obsessive-compulsive disorder^18^ was deemed ineligible because it pooled data only from two studies examining suicide death, both of which were clinical samples.

We synthesised individual-level risk factors for suicide mortality. Thus, we excluded meta-analyses of ecological factors such as lithium levels in drinking water^19^ and media reporting practices.^20^ Associations with biological, genetic, and perinatal factors were also beyond the scope of this study, which have been reviewed elsewhere.^21^, ^22^, ^23^, ^24^, ^25^ For psychiatric and physical health conditions, we only considered diagnoses according to standardised DSM or ICD criteria. Therefore, reviews examining drug use^26^, ^27^, ^28^, ^29^ or psychotic experiences^30^ without necessarily meeting diagnostic criteria were excluded, as was self-reported physical pain.^31^ Further, several meta-analyses published in 2022 were not eligible for inclusion because of conflating alcohol-related problems with alcohol use disorders,^32^ insufficient data on specific predictors relating to BMI,^33^ and the inclusion of mostly clinical samples in examining impulsivity and aggression.^34^ Preference was given to meta-analyses examining broad categories of risk factors (eg, occupational class by skill level was selected instead of specific occupational groups such as physicians^35^). If more than one eligible meta-analysis was identified on the same risk factor, the most recent one was included to avoid duplication of underlying samples.^9^ When multiple eligible meta-analyses on the same risk factor were published within a 2-year period, we retained the one with the largest pooled sample from the general population (appendix pp 9–10). For most risk factors, there were no marked differences in pooled estimates between these overlapping meta-analyses.

### Data extraction

Data were extracted independently by two investigators (including LF) using a standardised form. Discrepancies were resolved by discussion within the research team. For each eligible meta-analysis, we recorded the number and characteristics (eg, country, date of publication, and study design) of primary studies, study-specific risk estimates, and the pooled effect size (from random-effects models) with 95% CI and corresponding heterogeneity statistic (*I*^2^). An *I*^2^ value (which describes the percentage of variability in effect estimates that is due to between-study heterogeneity rather than chance) of less than 50% was taken to indicate low heterogeneity.^36^ Authors were contacted if study characteristics were unclear or when study-level estimates were not available in the paper. If reviews reported multiple levels of adjustment, we selected the estimates from the least adjusted model^6^ because this provided for the most comparable metric, as most studies did not provide adjusted estimates. When available, we extracted effect sizes for males and females separately. In case only sex-specific associations were reported for a given risk factor, we calculated an overall estimate (males and females combined) using random-effects models (appendix pp 22–23). Protective factors (ie, religious affiliation) were inversed so that all associations are reported in the same direction.^9^ Given the low incidence of suicide mortality in the general population,^1^ odds ratios, relative risks (or risk ratios), incidence rate ratios, hazard ratios, and standardised mortality ratios were treated as equivalent measures of risk.^9^

### Data analysis

Effect sizes were synthesised and compared across five risk factor domains: sociodemographic, physical, psychiatric, suicide-related, and other. Neurological disorders were included in the physical health domain. Three tests were conducted to assess the robustness and consistency of associations. First, based on the formula described by IntHout,^37^ we calculated 95% prediction intervals, which provide an estimate of the range in which future observations will fall. Risk factors with prediction intervals that cross the null value (eg, 1 in case of relative risk) were deemed to be of lower quality, indicating potentially non-significant findings if tested in a new population. Second, we assessed whether there was evidence for small-study effects (ie, whether smaller studies yield stronger associations relative to larger studies) using the regression asymmetry test proposed by Egger.^38^ A p value less than 0·10 was used to provide evidence for small-study effects. Third, the ratio between the pooled overall effect size of a meta-analysis and the effect size of its largest included study (assumed to be the most accurate) was calculated as a measure of statistical excess bias. A ratio greater than 1 is an indication of excess significance.^39^ For one risk factor, these tests could not be performed because only a pooled effect size (and no study-level estimates) was reported in the paper, and the authors did not respond to our multiple requests to provide such information.

The methodological quality of included meta-analyses was rated using the Risk of Bias in Systematic Reviews (ROBIS) instrument.^40^ ROBIS is a tool specifically designed to assess the risk of bias in systematic reviews, which is rated on four domains: study eligibility criteria (five items), identification and selection of studies (five items), data collection and study appraisal (five items), and synthesis and findings (six items). On the basis of these domain ratings, we computed an overall risk of bias score, classifying each review as having low, moderate, or high risk of bias (appendix p 11).

We used an overall quality assessment developed for umbrella reviews.^39^ Each identified risk factor was assigned a score of zero (low quality) or one (high quality) on five criteria: between-study heterogeneity, prediction intervals, small-study effects, excess significance, and risk of bias. The five quality scores were then summed to determine an aggregate quality rating within the range of zero to five, with zero designating the lowest overall quality and five designating the highest. Missing data on quality criteria were scored as zero. Composite scores of four or five (of five) indicate high-quality evidence for the respective risk factor.^39^

### Role of the funding source

The funder of the study had no role in study design, data collection, data analysis, data interpretation, or writing of the report.

## Results

Our systematic search of the literature yielded 3136 records for screening, of which 510 full-text reports were examined for eligibility (appendix p 24). We identified 79 meta-analyses that met our inclusion criteria, of which 46 were further excluded because they examined the same risk factor (appendix pp 12–14). No additional eligible studies were identified through manual and targeted searches. In total, we included data from 33 meta-analyses reporting on 38 risk factors for suicide mortality (appendix pp 15–16).^41^, ^42^, ^43^, ^44^, ^45^, ^46^, ^47^, ^48^, ^49^, ^50^, ^51^, ^52^, ^53^, ^54^, ^55^, ^56^, ^57^, ^58^, ^59^, ^60^, ^61^, ^62^, ^63^, ^64^, ^65^, ^66^, ^67^, ^68^, ^69^, ^70^, ^71^, ^72^, ^73^

Meta-analyses were published between 2008 and 2023, and the primary studies included in them dated from 1973 to 2021. The number of studies analysed in each meta-analysis ranged from four to 51 (with a mean of 14) and the number of individuals included ranged from 1069 to 117 million. Most meta-analyses (22 [67%] of 33) were restricted to data from high-income countries (HICs) and 11 (33%) also included primary studies from low-income and middle-income countries (LMICs). The proportion of primary studies from HICs was 93% (422/454) across all 33 meta-analyses and 84% (172/204)in the 11 meta-analyses that combined samples from HICs and LMICs (appendix p 17).

All meta-analyses included summary-level data from published literature (none pooled individual participant data) and all used random-effects models to calculate a pooled estimate. Although most (22 [67%] of 33) meta-analyses placed no restrictions on study designs, eight (24%) included only cohort studies and three (9%) only case-control studies. Several reviews specifically targeted psychological autopsy studies,^62^, ^72^ register-based studies,^67^ and prospective cohort studies.^44^, ^48^ In terms of outcome, 14 (42%) of 33 meta-analyses only focused on suicide mortality, whereas 19 (58%) additionally examined associations with suicide attempts, suicidal ideation, psychiatric disorders, or non-suicide mortality. One meta-analysis did not report study-level estimates of included primary studies,^54^ and 21 (64%) did not provide analyses stratified by sex. Definitions of the included risk factors are provided in the appendix (pp 18–19), all of which were operationalised dichotomously (present *vs* absent).

**Figure 1:**
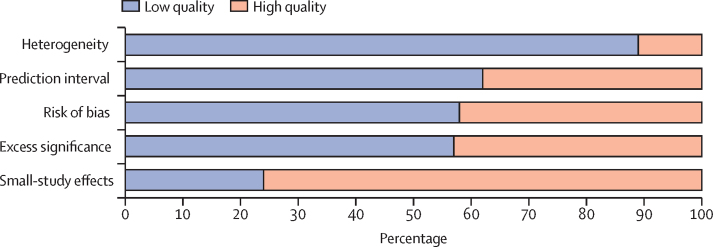
Overall quality assessment of risk factors

**Figure 2:**
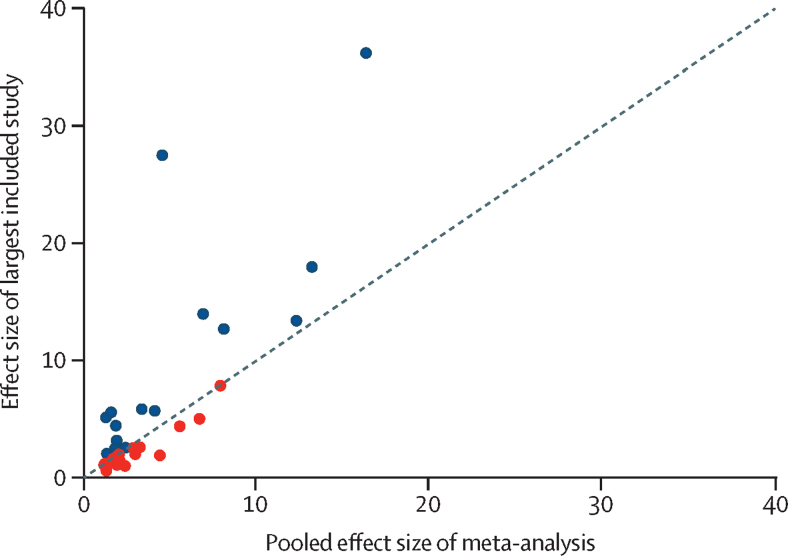
Excess significance in meta-analyses Points below the diagonal line (marked in red) indicate excess significance bias (ie, a larger pooled effect size in the meta-analysis relative to the effect size of its largest included study).

Based on ROBIS criteria, 12 (36%) of 33 of the meta-analyses were assessed as having an overall low risk of bias, 14 (42%) as moderate risk, and seven (21%) as high risk. Common limitations were the absence of a pre-registered study protocol (55% [18/33]), risk of bias assessment (30% [10/33]), or meta-regression to examine sources of heterogeneity (52% [17/33]), and the absence of independent data extraction by multiple reviewers (36% [12/33]). As two meta-analyses^55^, ^67^ provided data for multiple risk factors, 22 (58%) of 38 risk estimates were based on meta-analyses with moderate or high risk of bias (table). Other quality tests performed at the risk factor level also found indications of poor quality (figure 1). Heterogeneity was high (*I*^2^>50%) for 89% (34/38) of risk factor estimates. Of the 37 risk factors for which sufficient data were available, 23 (62%) had 95% prediction intervals that included the null value, 21 (57%) showed evidence of excess significance (figure 2), and nine (24%) had small-study effects. Overall quality ratings indicated variable but mostly low quality. Using a scoring system based on five quality indicators, the mean score across 38 risk factors was two (of five). No risk factor met full criteria. Six (16%) risk factors had high quality scores, mostly within the psychiatric domain (ie, psychotic disorders, mood disorders, personality disorders, anorexia nervosa, smoking, and state care in childhood). With composite scores of two or less, quality was low for 68% (26/38) of all risk factors examined. By domain, overall quality scores were especially low for physical health and sociodemographic factors.

**Table:** Risk factors for suicide mortality, by domain

	**k**	**Statistic**	**Effect size (95% CI)**	**I^2^**	**Prediction interval**	**Small-study effects (p)**	**Excess significance**	**Risk of bias**	**Overall quality**
**Physical domain**									
Epilepsy^58^	6	RR	2·9 (2·2–3·8)	52%	1·5–5·6	0·198	1·1	Moderate	2
Hidradenitis suppurativa^60^	4	OR	2·1 (1·3–3·4)	98%	0·4–11·9	0·673	1·4	High	1
Concussion^50^	6	RR	2·0 (1·5–2·8)	96%	0·7–5·7	0·526	1·0	Low	2
COPD^63^	5	OR	1·9 (1·3–2·9)	95%	0·5–7·5	0·107	0·6	Moderate	2
Cancer^51^	28	SMR	1·9 (1·6–2·2)	99%	0·8–4·1	0·245	0·4	Low	3
Multiple sclerosis^65^	16	IRR	1·7 (1·4–2·2)	87%	0·7–4·1	0·673	1·1	Moderate	1
Stroke^69^	14	RR	1·6 (1·4–1·8)	92%	0·9–2·7	0·690	1·2	Moderate	1
Parkinson's disease^47^	4	SMR	1·6 (1·3–1·9)	89%	0·2–12·1	0·021	0·3	High	1
Diabetes^70^	28	RR	1·6 (1·3–1·9)	93%	0·6–3·9	0·483	1·2	Low	2
Psoriasis^53^	7	HR	1·3 (0·9–2·0)	87%	0·4–4·4	0·808	0·7	Moderate	2
Asthma^73^	8	OR	1·3 (1·1–1·6)	80%	0·8–2·2	0·303	1·1	Moderate	1
Dementia^41^	16	OR	1·3 (0·8–2·1)	96%	0·2–10·1	0·148	0·3	Moderate	2
Inflammatory bowel disease^71^	17	RR	1·3 (1·1–1·4)	56%	0·8–2·0	0·841	1·0	Moderate	1
**Psychiatric domain**									
Psychotic disorders^67^	4	RR	13·2 (8·6–20·3)	98%	3·4–51·8	0·292	0·7	Low	4
Mood disorders^67^	7	RR	12·3 (8·9–17·1)	97%	4·2–36·4	0·463	0·9	Low	4
Personality disorders^67^	3	RR	8·1 (4·6–14·2)	94%	1·5–45·0	0·549	0·6	Low	4
Anorexia nervosa^49^	9	RR	6·9 (4·1–11·5)	0%	3·7–12·9	0·269	0·5	Moderate	4
ADHD^64^	4	OR	6·7 (3·2–13·8)	88%	0·7–62·9	0·716	1·3	Low	2
Substance use disorders^67^	6	RR	4·4 (2·9–6·8)	98%	1·1–18·5	0·370	2·3	Low	3
Anxiety disorders^67^	6	RR	4·1 (2·4–6·9)	98%	0·5–31·2	0·310	0·7	Low	3
**Suicide-related domain**									
Suicide attempt/self-harm^72^	11	OR	16·3 (7·5–35·5)	74%	1·3–208·7	0·028	0·5	High	2
Suicidal ideation^53^	14	RR	5·6 (3·1–10·1)	79%	0·8–41·2	0·044	1·3	Low	1
Parental suicide^45^	10	RR	3·0 (2·5–3·5)	85%	1·6–5·5	0·006	1·5	Low	2
**Sociodemographic domain**									
Unsecured financial debt^62^	4	OR	7·9 (5·2–12·0)	0%	4·0–15·6	0·513	1·0	High	3
No religious affiliation^61^	14	RR	2·4 (1·9–2·9)	94%	1·23–4·6	0·186	2·4	High	2
Low education^55^	4	RR	1·9 (1·2–3·0)	74%	0·5–7·3	0·010	1·8	High	0
Unemployment^42^	21	OR	1·9 (1·4–2·5)	100%	0·2–18·4	0·496	1·1	High	1
Low income^55^	4	RR	1·8 (1·3–2·5)	91%	0·7–4·9	0·045	0·7	High	1
Low skill level occupation^56^	34	IRR	1·8 (1·4–2·2)	99%	0·3–10·7	0·055	1·1	Moderate	0
Not married^54^*	36	OR	1·6 (1·4–1·9)	99%	..	..	..	Low	1
Ethnic minority^68^	51	IRR	1·3 (0·9–1·7)	100%	0·1–83·8	0·352	2·2	Low	2
Job stressors^57^	6	OR	1·2 (1·0–1·3)	96%	0·7–1·9	0·031	1·1	Moderate	0
**Other domain**									
Criminal offending^66^	15	OR	4·5 (2·1–9·7)	100%	1·6–12·9	0·572	0·2	Moderate	3
State care in childhood^44^	4	RR	3·4 (2·4–4·7)	72%	1·2–9·0	0·790	0·6	Low	4
Access to firearms^43^	14	OR	3·2 (2·4–4·4)	89%	1·2–8·9	0·897	1·3	Low	3
Smoking^48^	15	RR	2·4 (2·1–2·8)	49%	1·5–3·8	0·673	0·9	Moderate	4
Sleep disturbances^46^	5	RR	1·8 (1·3–2·4)	59%	0·8–4·1	0·079	0·9	Low	2
BMI (underweight)^59^	9	HR	1·2 (1·1–1·4)	38%	0·9–1·6	0·230	1·0	Moderate	2

Overall quality scores range from 0 to 5, with higher scores indicating higher quality. ADHD=attention-deficit hyperactivity disorder. COPD=chronic obstructive pulmonary disease. HR=hazard ratio. IRR=incidence rate ratio. OR=odds ratio. RR=relative risk or risk ratio. SMR=standardised mortality ratio.

* Quality tests could not be performed because no study-level data were available.

**Figure 3:**
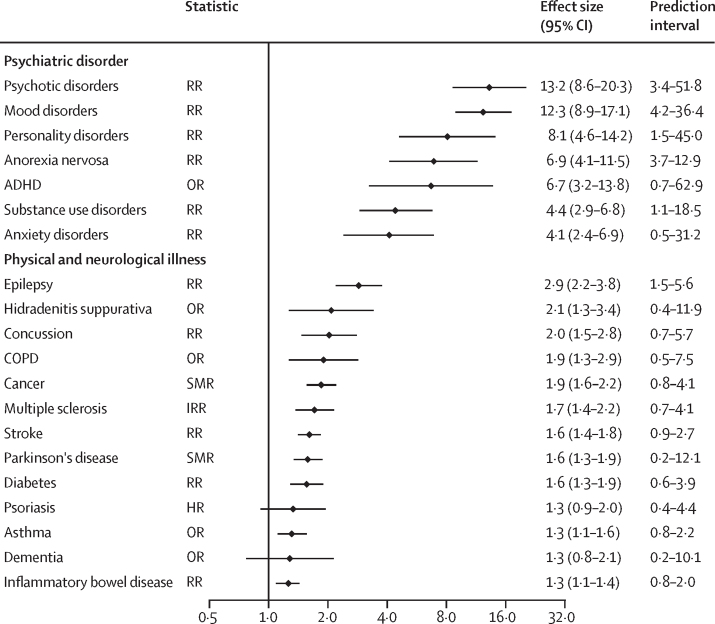
Risk factors for suicide mortality in the psychiatric and physical health domains ADHD=attention-deficit hyperactivity disorder. COPD=chronic obstructive pulmonary disease. HR=hazard ratio. IRR=incidence rate ratio. OR=odds ratio. RR=relative risk or risk ratio. SMR=standardised mortality ratio.

Suicide mortality was significantly associated with all but three (dementia, psoriasis, and ethnic minority) investigated risk factors, with effect sizes ranging from 1·2 (95% CI 1·0–1·3) for job stressors to 16·3 (7·5–35·5) for suicide attempts and self-harm (table). By domain, the strongest associations with suicide mortality were found for psychiatric (effect size range 4–13; figure 3) and suicide-related (range 3–16) risk factors. Mood disorders and psychotic disorders (including schizophrenia) had the largest effect sizes within the psychiatric domain, and previous suicide attempt or self-harm was the strongest risk factor overall. Within the physical health domain, epilepsy, concussion, chronic obstructive pulmonary disease, and cancer had strong links with suicide risk (figure 3). Sociodemographic factors were moderately associated with suicide mortality, with unsecured financial debt being an outlier (which was based on only four psychological autopsy studies). Across 13 risk factors for which sex-specific estimates were available, effect sizes were largely comparable for males and females, with overlapping CIs except for marital status (appendix p 20). The effects of income and education on suicide mortality were significant for males but not for females, albeit with overlap in their CIs.

For meta-analyses that additionally examined outcomes other than suicide mortality, we have presented the association of risk factors with these outcomes (suicide attempt, suicidal ideation, and non-suicide mortality) in the appendix (p 21). There were higher relative risks for dementia and sleep disturbances for suicide attempts than suicide mortality, but lower for parental death by suicide and religious affiliation (with non-overlapping CIs) and for ADHD and smoking (with overlapping CIs).

## Discussion

We have presented an overview of 38 risk factors for suicide mortality reported in 33 meta-analyses based on research over five decades and have appraised the quality of the evidence. Associations should be interpreted in light of the high heterogeneity between primary studies and excess statistical significance.

A wide range of contributory factors were identified across various domains, underscoring death by suicide as a multifactorial phenomenon.^2^, ^3^, ^4^ By domain, we found marked differences between risk factors in their strength of association with suicide mortality. First, the strongest associations were found in the psychiatric and suicide-related domains. Large effect sizes for a previous suicide attempt and suicidal ideation support a continuum approach to suicide risk,^74^ and the relationship with parental death by suicide points to familial transmission of risk^75^ (eg, through genetic liability, social learning, or shared exposure to adverse environments). Psychiatric disorders, such as mood and psychotic disorders, increase the risk of suicide mortality approximately 10-fold, and this was supported by high-quality evidence. This finding reinforces the need to focus on these high-risk populations in primary and secondary care. Clinical guidelines should incorporate the identification, assessment, and treatment of psychiatric disorders.^76^ Psychosocial interventions following self-harm, including cognitive behavioural therapy, might reduce suicide risk.^77^ There was also an elevated risk for several physical illnesses—such as cancer and epilepsy—but at considerably lower levels of risk than for psychiatric disorders, with effect sizes of approximately two (for which the quality of evidence was not high). It is important for clinicians in primary and secondary care to consider the wider effects of physical illnesses and to liaise with mental health services to address suicide risk. Training general practitioners and health-care professionals is a promising strategy,^78^ as most individuals who die by suicide, at least in high-income settings, make contact with primary care in the year before death.^79^ More than 90% of primary studies included in the meta-analyses were from HICs, and two thirds of all meta-analyses did not include any data from LMICs, where nearly 80% of all suicide deaths worldwide occur.^80^ A better understanding of the risk factors for suicide mortality in LMICs is important to ensure that the limited resources in LMICs are effectively targeted and directed to the appropriate services and prevention interventions. If the findings by domain are replicated in LMICs, one key implication would be to improve access to mental health services in LMICs.

Second, various risk factors were identified in the non-clinical domains. Although sociodemographic factors were only moderately associated with suicide mortality and based on mostly low-quality evidence, these factors could be important as they can be prevalent at specific periods and for particular age groups (eg, financial debt and unemployment).^81^ Innovative public health strategies, which would require working with policy makers and across local and national government, will be needed. The wider social determinants of health should be addressed by a whole-of-government approach.^82^ The link with access to firearms further underscores that means restriction should be part of any national prevention strategy.^78^ Furthermore, there are high-risk groups identified in our review, such as people in contact with the criminal justice system and those who experienced state care in childhood—underscoring the need for a life-course perspective to understanding suicide.^83^ Other public health interventions include substance misuse policies, with a role for improving access to addiction services and strengthening restrictive alcohol policies, which could decrease suicide risk at the population level.^84^

Across risk factors for which sex-stratified estimates were available, associations were largely similar for males and females, but approximately two-thirds of meta-analyses did not provide such information. Further examination of differences and similarities in associations with suicide mortality between males and females could advance our understanding of sex-specific risk factors. In a secondary analysis, we further found that several risk factors (eg, sleep disturbances and parental suicide) had notable differences in their strength of association with suicide mortality compared with attempted suicide, suggesting that these are distinct outcomes.

Four points should be considered when interpreting the risk factor estimates presented in this review. First, effect sizes across individual meta-analyses might not be strictly comparable due to varying levels of adjustment and different study designs. For example, some meta-analyses were restricted to record linkage studies^67^ or psychological autopsy investigations,^62^, ^72^ which makes head-to-head comparisons of effect sizes difficult. Second, the identified risk factors—each one examined in a separate meta-analysis—might not be independent of each other, and thus the effects will not be cumulative. How the risk factors interact needs to be considered. Delineating independent associations at the meta-analytic level would require pooling individual participant data using multivariable models, but we did not identify any such initiatives in this area. Third, due to unmeasured confounding, the observational nature of primary studies included in the meta-analyses does not allow us to establish a causal link between risk factors and suicide mortality.^85^ Evidence of causal inference will need to be tested in high-quality studies such as from sibling control designs, Mendelian randomisation, natural experiments, and, when feasible, trials. Fourth, the reported effect sizes do not quantify the effect of risk factors at the population level. Policy planning needs to consider population attributable fractions, which take into account both the prevalence of a risk factor and the magnitude of its effect, assuming causality. One example is a meta-analysis estimating that a fifth of suicide deaths would be prevented if exposure to mood disorders were to be eliminated in the population.^67^

In addition to providing an overview of risk factors, we evaluated whether there were indications of bias in this meta-analytic literature. We found that the overall quality of the evidence was not strong, with most reported evidence on risk factors showing high heterogeneity and excess significance. For 62% of identified risk factors, prediction intervals included the null value, suggesting poor replicability in future studies. Some of the reported associations with suicide mortality should therefore be interpreted with caution, and effect size ranges should be considered. Furthermore, only 36% of included meta-analyses were rated as low risk of bias. Common limitations included the absence of pre-registered protocols and insufficient consideration of heterogeneity and bias in primary studies. Future reviews should therefore focus on adhering to methodological guidelines.^10^ Other recommendations for future meta-analyses include considering confounding more carefully, reporting pooled analyses disaggregated by sex, and estimating population attributable fractions for the risk factors being investigated.

Strengths of this umbrella review include synthesising a broad range of risk factors from published meta-analyses, comparing effect sizes across domains, and using tests of methodological quality to determine the robustness and consistency of the evidence. However, there are also several limitations. The validity of any umbrella review depends on the coverage and quality of both the meta-analyses and their primary studies. Our findings only apply to suicide mortality in the general population and might not be generalisable to clinical and other high-risk populations, in which effect sizes are likely to be smaller.^6^ Furthermore, the overview of risk factors presented was determined by our inclusion criteria in three ways. First, we did not consider biological, genetic, perinatal, and ecological factors, as these were outside the study scope and are not directly comparable with individual-level factors examined in the current review. Second, as we only considered factors that have been subject to meta-analysis, potential risk factors examined in narrative or systematic reviews (without quantitative synthesis) were not discussed. Among others, these risk factors include childhood adversity, social isolation, homelessness, interpersonal violence, intellectual disability, and autism spectrum disorder. These gaps highlight areas where meta-analytic approaches would be useful, provided that it is appropriate to pool such evidence. Third, as we aimed to present a comprehensive synthesis of the evidence, we excluded meta-analyses that only included data on specific suicide methods and individual countries, as these would probably limit generalisability. Another consideration relates to our selection of risk factor estimates. For psychiatric disorders, we considered diagnostic categories rather than specific diagnoses. However, differences in risk estimates within diagnostic categories have been reported (eg, dysthymia *vs* major depression for mood disorders, with similar differences for personality disorders).^4^ In addition, when multiple meta-analyses presented overlapping data on the same risk factor, a single review was selected to avoid duplication, but the choice of review could have influenced findings (although effect sizes were not materially different). We retained the most recent meta-analysis to capture the largest evidence base; however, older reviews might have been of higher quality. Although we did not use a citation matrix to quantify the degree of overlap,^86^ we observed striking discrepancies based on which primary studies were included in different meta-analyses examining the same risk factor, despite being published in the same period and adopting similar inclusion criteria. Two recent reviews highlight this issue examining the association between cancer and suicide.^8^, ^51^ Finally, we did not assess the quality of primary studies included in meta-analyses, nor did we re-analyse each meta-analysis with individual study estimates^39^ or examine trends over time in terms of risk factor coverage or quality.

In conclusion, we have systematically assessed the strength and quality of the meta-analytic evidence for 38 risk factors for suicide mortality. It is notable that most of the identified risk factors are modifiable. Individual and population-based prevention strategies should account for these factors and their relative strengths. Public health interventions, spanning primary and secondary care, improving liaison between physical and mental health services, and targeting high-risk groups (including people who have previously attempted suicide, those in contact with the criminal justice system, and individuals who have recently incurred unsecured debt) should be further developed. On the other hand, the quality of the meta-analytic evidence is limited by excess significance and high heterogeneity, and prediction intervals suggest poor replicability. Given the major burden of suicide mortality on public health, a focus on higher quality research is needed to inform suicide prevention efforts.

## Data sharing

Study data are available from the corresponding author upon request.

## Declaration of interests

We declare no competing interests.
