# Supplementary material for: Individual-level risk factors for suicide mortality in the general population: an umbrella review
Source: Lancet Public Health. 2023 Oct 26;8(11):e868–77. doi: 10.1016/S2468-2667(23)00207-4 (PMC10932753; doi:10.1016/S2468-2667(23)00207-4)
Supplement: Supplementary appendix [file mmc1.pdf]

THE LANCET
Public Health

Supplementary appendix

This appendix formed part of the original submission and has been peer reviewed. We post it as supplied by the authors.

Supplement to: Favril L, Yu R, Geddes JR, Fazel S. Individual-level risk factors for suicide mortality in the general population: an umbrella review. *Lancet Public Health* 2023;
8: e868–77.

Supplementary materials

Table S1. PRISMA checklist.

Table S2. Summary of findings from the meta-analyses by Franklin and colleagues.

Table S3. Selection of overlapping eligible meta-analyses.

Table S4. ROBIS scoring system.

Table S5. Overlapping meta-analyses that were excluded, by domain.

Table S6. Characteristics of included meta-analyses.

Table S7. Included primary studies by county income level.

Table S8. Definition of exposures.

Table S9. Risk factors for suicide stratified by sex.

Table S10. Meta-analyses that additionally examined associations with other outcomes.

Figure S1. Association between income and suicide, men and women combined.

Figure S2. Association between education and suicide, men and women combined.

Figure S3. Study selection.

Supplementary references.

Table S1. PRISMA checklist.

Section and Topic	Item #	Checklist item	Location where item is reported
**TITLE**			
Title	1	Identify the report as a systematic review.	NA
**ABSTRACT**			
Abstract	2	See the PRISMA 2020 for Abstracts checklist.	p2
**INTRODUCTION**			
Rationale	3	Describe the rationale for the review in the context of existing knowledge.	p1
Objectives	4	Provide an explicit statement of the objective(s) or question(s) the review addresses.	p1
**METHODS**			
Eligibility criteria	5	Specify the inclusion and exclusion criteria for the review and how studies were grouped for the syntheses.	p4-6
Information sources	6	Specify all databases, registers, websites, organisations, reference lists and other sources searched or consulted to identify studies. Specify the date when each source was last searched or consulted.	p4
Search strategy	7	Present the full search strategies for all databases, registers and websites, including any filters and limits used.	p4
Selection process	8	Specify the methods used to decide whether a study met the inclusion criteria of the review, including how many reviewers screened each record and each report retrieved, whether they worked independently, and if applicable, details of automation tools used in the process.	p5
Data collection process	9	Specify the methods used to collect data from reports, including how many reviewers collected data from each report, whether they worked independently, any processes for obtaining or confirming data from study investigators, and if applicable, details of automation tools used in the process.	p6
Data items	10a	List and define all outcomes for which data were sought. Specify whether all results that were compatible with each outcome domain in each study were sought (e.g. for all measures, time points, analyses), and if not, the methods used to decide which results to collect.	p5
	10b	List and define all other variables for which data were sought (e.g. participant and intervention characteristics, funding sources). Describe any assumptions made about any missing or unclear information.	p5
Study risk of bias assessment	11	Specify the methods used to assess risk of bias in the included studies, including details of the tool(s) used, how many reviewers assessed each study and whether they worked independently, and if applicable, details of automation tools used in the process.	p7
Effect measures	12	Specify for each outcome the effect measure(s) (e.g. risk ratio, mean difference) used in the synthesis or presentation of results.	p6

Synthesis methods	13a	Describe the processes used to decide which studies were eligible for each synthesis (e.g. tabulating the study intervention characteristics and comparing against the planned groups for each synthesis (item #5)).	p6
	13b	Describe any methods required to prepare the data for presentation or synthesis, such as handling of missing summary statistics, or data conversions.	p6
	13c	Describe any methods used to tabulate or visually display results of individual studies and syntheses.	NA
	13d	Describe any methods used to synthesize results and provide a rationale for the choice(s). If meta-analysis was performed, describe the model(s), method(s) to identify the presence and extent of statistical heterogeneity, and software package(s) used.	p6-7
	13e	Describe any methods used to explore possible causes of heterogeneity among study results (e.g. subgroup analysis, meta-regression).	NA
	13f	Describe any sensitivity analyses conducted to assess robustness of the synthesized results.	NA
Reporting bias assessment	14	Describe any methods used to assess risk of bias due to missing results in a synthesis (arising from reporting biases).	p7
Certainty assessment	15	Describe any methods used to assess certainty (or confidence) in the body of evidence for an outcome.	p7
**RESULTS**			
Study selection	16a	Describe the results of the search and selection process, from the number of records identified in the search to the number of studies included in the review, ideally using a flow diagram.	p8, figure S3
	16b	Cite studies that might appear to meet the inclusion criteria, but which were excluded, and explain why they were excluded.	p5
Study characteristics	17	Cite each included study and present its characteristics.	p8, table S6
Risk of bias in studies	18	Present assessments of risk of bias for each included study.	p8, figure 1
Results of individual studies	19	For all outcomes, present, for each study: (a) summary statistics for each group (where appropriate) and (b) an effect estimate and its precision (e.g. confidence/credible interval), ideally using structured tables or plots.	Table 1
Results of syntheses	20a	For each synthesis, briefly summarise the characteristics and risk of bias among contributing studies.	p8-9
	20b	Present results of all statistical syntheses conducted. If meta-analysis was done, present for each the summary estimate and its precision (e.g. confidence/credible interval) and measures of statistical heterogeneity. If comparing groups, describe the direction of the effect.	p9, table 1
	20c	Present results of all investigations of possible causes of heterogeneity among study results.	NA
	20d	Present results of all sensitivity analyses conducted to assess the robustness of the synthesized results.	NA
Reporting biases	21	Present assessments of risk of bias due to missing results (arising from reporting biases) for each synthesis assessed.	Table 1
Certainty of evidence	22	Present assessments of certainty (or confidence) in the body of evidence for each outcome assessed.	p8-9

**DISCUSSION**			
Discussion	23a	Provide a general interpretation of the results in the context of other evidence.	p9-10
	23b	Discuss any limitations of the evidence included in the review.	p11
	23c	Discuss any limitations of the review processes used.	p12
	23d	Discuss implications of the results for practice, policy, and future research.	p10-11
**OTHER INFORMATION**			
Registration and protocol	24a	Provide registration information for the review, including register name and registration number, or state that the review was not registered.	p4
	24b	Indicate where the review protocol can be accessed, or state that a protocol was not prepared.	p4
	24c	Describe and explain any amendments to information provided at registration or in the protocol.	NA
Support	25	Describe sources of financial or non-financial support for the review, and the role of the funders or sponsors in the review.	p13
Competing interests	26	Declare any competing interests of review authors.	p13
Availability of data, code and other materials	27	Report which of the following are publicly available and where they can be found: template data collection forms; data extracted from included studies; data used for all analyses; analytic code; any other materials used in the review.	p13

Table S2. Summary of findings from the meta-analyses by Franklin and colleagues.

Meta-analysis/risk factor	OR (95% CI) for suicide	% community samples*
**Bentley et al. (2016)**		30.4
Any anxiety symptoms/diagnosis	1.01 (0.87–1.18)	
Anxiety symptoms	1.20 (0.95–1.52)	
Anxiety diagnosis	0.93 (0.77–1.13)	
Adjustment disorder	0.24 (0.09–0.67)	
Obsessive-compulsive disorder	0.23 (0.03–1.69)	
Panic disorder	1.09 (0.39–3.04)	
**Chang et al. (2016)**		43.6†
Biological risk factors	Not eligible	
**Franklin et al. (2017)**		40.4
Prior psychiatric hospitalisation	3.57 (2.81–4.53)	
Prior suicide attempt	2.24 (1.69–2.97)	
Prior suicide ideation	2.22 (1.45–3.41)	
Low socioeconomic status	2.20 (1.32–3.67)	
Stressful life events	2.18 (1.63–2.93)	
**Glenn et al. (2018)**		Not reported
Overall RDoC domains	1.41 (1.24–1.60)	
Arousal and regulatory systems	1.38 (1.12–1.70)	
Arousal	0.91 (0.54–1.53)	
Sleep-wakefulness	1.55 (1.33–1.80)	
Biological risk factors	1.96 (1.50–2.55)	
Dopaminergic function	1.03 (0.43–2.46)	
Neuroendocrine function	1.76 (1.17–2.66)	
Serotonergic function	1.10 (0.53–2.25)	
Cognitive Systems	0.96 (0.53–1.74)	
Cognitive control	0.98 (0.49–1.95)	
Negative valence systems	1.61 (1.40–1.86)	
Frustrative non-reward	1.89 (1.51–2.38)	
Loss: depressed mood	2.16 (1.19–3.93)	
Loss: guilt	1.39 (0.61–3.17)	
Loss: hopelessness	2.15 (1.53–3.02)	
Loss: rumination	2.16 (1.28–3.66)	
Neuroticism	1.42 (0.92–2.20)	
Potential threat	1.08 (0.72–1.60)	
Sustained threat	1.24 (0.91–1.68)	
Positive valence systems	0.50 (0.17–1.46)	
Systems for social processes	1.06 (0.68–1.65)	
Affiliation and attachment	1.10 (0.77–1.57)	
Perception and understanding of self	0.34 (0.01–9.20)	
**Harris et al. (2020)**		48.1†
Insomnia	1.54 (1.04–2.29)	
Nightmares	1.31 (0.83–2.06)	

Sleep problems	1.27 (0.97–1.66)	
**Harris et al. (2022)**		94.7[†]
Body mass index	1.00 (0.78–1.30)	
**Huang et al. (2017)**		54.7
Demographics	1.34 (1.18–1.52)	
Age	1.09 (0.97–1.24)	
Sex	1.50 (1.24–1.82)	
Race/ethnicity	1.70 (1.09–2.67)	
Family types	0.93 (0.65–1.33)	
Education level	1.34 (1.27–1.42)	
Employment status	1.41 (1.05–1.90)	
Socioeconomic status	2.65 (1.52–4.63)	
Marital status	1.62 (1.34–1.95)	
Single	2.24 (1.81–2.77)	
Married	0.76 (0.33–1.70)	
Divorced	0.97 (0.19–4.98)	
**Huang et al. (2018)**		10.9
Any psychosis diagnosis/symptoms	1.40 (1.14–1.72)	
Psychosis diagnosis	1.71 (1.30–2.26)	
Schizophrenia	1.62 (0.90–2.93)	
Schizoaffective disorder	1.50 (0.71–3.16)	
Unspecified psychosis diagnosis	2.09 (1.54–2.84)	
Psychosis symptoms	1.14 (0.79–1.66)	
Overall symptoms	2.11 (1.12–3.98)	
Positive symptoms	1.59 (1.17–2.16)	
Negative symptoms	0.30 (0.15–0.60)	
**Huang et al. (2020)**		40.3[†]
Brain differences	Not eligible	
**Ribeiro et al. (2016)**		5.1
Suicidal ideation	1.95 (1.31–2.90)	
Suicide plans	1.44 (1.11–1.86)	
Suicide attempt	2.03 (1.61–2.57)	
Suicide attempt features	1.30 (1.18–1.43)	
Concerning reaction	1.86 (0.99–3.50)	
Intoxication	0.92 (0.58–1.46)	
Intent	1.20 (1.09–1.31)	
Lethality	1.17 (0.91–1.50)	
Number	1.28 (0.87–1.88)	
Preparations	1.28 (0.96–1.71)	
Violent attempt	1.89 (1.21–2.97)	
Self-injurious thoughts and behaviours	2.28 (1.68–3.11)	
Deliberate self-harm	1.51 (1.13–3.01)	
Family history of SITB	1.63 (0.93–2.84)	
**Ribeiro et al. (2018)**		17.6
Hopelessness	1.98 (1.46–1.69)	

Major depressive disorder diagnosis	1.50 (1.04–2.17)	
Depression symptoms	1.28 (1.16–1.41)	
Self-report inventories	1.07 (0.77–1.49)	
Beck Depression Inventory	1.04 (0.63–1.71)	
Clinical features of depression	1.00 (0.99–1.01)	
Number of major depressive episodes	0.83 (0.60–1.15)	
Major depressive disorder type	1.45 (1.08–1.96)	
Onset type	1.07 (0.80–1.43)	
Unspecified mood disorder	1.64 (1.11–2.42)	
**Smith et al. (2019)**		38.5
Eating disorder diagnosis	1.71 (0.98–2.98)	
**Witte et al. (2018)**		56.1[†]
Alcohol	1.65(0.99–2.75)	
Alcohol use	1.61(0.74–3.50)	
Alcohol frequency/quantity	1.60 (0.71–3.62)	
Alcohol use disorder	1.68 (0.84–3.33)	
Alcohol use disorder	1.38 (0.36–5.35)	
Alcohol abuse	1.86 (0.57–6.09)	
Problems related to alcohol	1.77 (0.51–6.10)	
Antisocial behaviour/conduct problems	2.50 (1.43–4.36)	
Aggression	2.21 (0.69–7.12)	
Antisocial behaviour	2.22 (1.06–4.66)	
Antisocial behaviour	1.99 (0.38–10.55)	
Violent antisocial behaviour	3.34 (1.14–9.79)	
Involvement with justice system	3.97 (1.16–13.55)	
Cognitive/emotional/personality traits	1.34 (0.62–2.98)	
Disinhibition	0.69 (0.15–3.09)	
Hostility	1.93 (0.55–6.78)	
Impulsivity	1.49 (0.41–5.39)	
Drugs	1.40 (0.85–2.31)	
Drug/alcohol use disorder	1.30 (0.34–4.96)	
Drug use	2.20 (0.87–5.55)	
Drug use	1.91 (0.45–8.12)	
Drug quantity/frequency	2.45 (0.66–9.11)	
Drug use disorder	0.70 (0.28–1.73)	
Drug abuse	0.48 (0.16–1.49)	
Marijuana use	1.53 (0.33–7.07)	
Marijuana use	1.52 (0.30–7.66)	
Smoking	1.82 (1.21–2.73)	
Current smoking	2.08 (1.28–3.38)	
Current smoking	1.95 (1.09–3.49)	
Current dependence/heavy smoking	2.64 (0.89–7.86)	
Lifetime smoking	1.33 (0.64–2.79)	
Lifetime smoking	1.33 (0.61–2.92)	

* The authors of these meta-analyses classified primary studies into three categories based on sample type. A sample was coded as *self-injurious* when the entire sample was drawn based on a history of previous self-injurious thoughts and behaviours. A sample was defined as *clinical* if participants were selected based on clinical conditions (e.g., a diagnosis or score above a predetermined threshold on a clinical measure). When neither condition was met, a sample was coded as a *community* sample (i.e., general population). Percentages listed are for community samples (specifically examining *suicide death* as outcome, unless specified otherwise). In the overarching meta-analysis by Franklin et al. (2017), analyses showed that the magnitude of odds ratios for suicide death declined from community samples to clinical and self-injurious samples.

† Numbers listed refer to the percentage of community samples across all three different outcomes examined (suicidal ideation, suicide attempt, and suicide death combined) because no figures were reported specifically for death by suicide.

Table S3. Selection of overlapping eligible meta-analyses.

Risk factor/meta-analysis	Studies included	Pooled sample	Effect size (95% CI)
**Suicidal ideation**[*]			
Hubers et al. (2018)	81	4,601,378	RR = 4.17 (3.29–5.27)
General population	14	145,411	RR = 5.55 (3.06–10.1)
McHugh et al. (2019)	71	4,669,303	OR = 3.41 (2.59–4.49)
**Sleep disturbances**			
Liu et al. (2020)	14	664,141	d = 0.50 (0.14–0.85)[†]
Dong et al. (2021)	5	1,088,983	RR = 1.80 (1.32–2.44)
**Diabetes**			
Wang et al. (2016)	6	3,075,214	RR = 1.61 (0.91–2.83)
Wang et al. (2017)	28	28,057,822	RR = 1.56 (1.29–1.89)
Elamoshy et al. (2018)	5	Not reported	OR = 1.85 (0.97–3.52)
**Body mass index**			
Perera et al. (2016)	15	10,130,420	—
Underweight	9	9,122,396	HR = 1.21 (1.07–1.36)
Overweight	7	6,209,202	HR = 0.78 (0.75–0.82)
Obesity	6	6,162,447	HR = 0.71 (0.56–0.89)
Amiri & Behnezhad (2018)	10	3,854,569	—
Overweight	9	3,841,681	RR = 0.78 (0.71–0.85)
Obesity	9	3,844,331	RR = 0.67 (0.54–0.81)
**Cancer**			
Amiri & Behnezhad (2020)	22	Not reported	SMR = 1.55 (1.37–1.74)
Ravaioli et al. (2020)	20	Not reported	SMR = 1.70 (1.50–1.90)
Heinrich et al. (2022)	28	22,407,690	SMR = 1.85 (1.55–2.20)
**Suicide exposure**[‡]			
Hill et al. (2020)	16	13,466,328	—
Suicide	11	13,464,582	OR = 3.23 (2.32–4.51)
Suicide attempt	3	723	OR = 1.64 (0.90–2.98)
Suicidal behaviour	4	1479	OR = 3.83 (2.38–6.17)
Calderaro et al. (2022)	10	17,483,726	RR = 2.97 (2.50–3.53)
**Ethnic minority**			
Amiri (2022)	19	Not reported	OR = 1.10 (1.08–1.11)
Troya et al. (2022)	51	1,028,561	RR = 1.30 (0.90–1.70)
**Anorexia nervosa**			
Ferrari et al. (2014)	9	Not reported	RR = 6.9 (4.1–11.5)
Keshaviah et al. (2014)	39	Not reported	SMR = 18.1 (11.5–28.7)
**Psychiatric disorders**[§]			
Conner et al. (2019)	35	5594	—
Mood disorder	18	2953	OR = 14.34 (9.10–22.57)
Minor depression	12	1446	OR = 2.73 (1.53–4.85)
Major depression	19	2351	OR = 9.14 (5.53–15.09)
Bipolar disorder	8	1291	OR = 3.70 (1.59–8.61)
Substance use disorder	20	3520	OR = 4.09 (3.10–5.40)

Drug use disorder	8	1142	OR = 7.18 (3.22–16.01)
Alcohol use disorder	11	1549	OR = 3.68 (1.99–6.82)
Too et al. (2019)	13	6,475,685	—
Mood disorders	7	5,584,409	RR = 12.3 (8.9–17.1)
Anxiety disorders	6	5,432,246	RR = 4.1 (2.4–6.9)
Psychotic disorders	4	667,171	RR = 13.2 (8.6–20.3)
Substance use disorders	6	2,036,063	RR = 4.4 (2.9–6.8)
Personality disorders	3	107,833	RR = 8.1 (4.6–14.2)
Moitra et al. (2021)	20	18,000	—
Major depression	14	11,736	RR = 7.64 (4.30–13.58)
Dysthymia	4	1753	RR = 4.11 (2.09–8.09)
Bipolar disorder	7	3333	RR = 6.05 (3.38–10.83)
Schizophrenia	13	9319	RR = 5.98 (3.33–10.72)
Anxiety disorders	17	12,583	RR = 4.89 (2.76–8.69)

Note. Data refer to suicide mortality. The selected meta-analysis/risk estimate is highlighted in grey.

* Of the 71 studies included in the meta-analysis by McHugh et al. (2019), 58 (82%) were of current or former psychiatric patients, including 42 studies of psychiatric inpatients. In subgroup analyses, the authors found that studies conducted in non-psychiatric settings (including primary healthcare, general population samples, military populations, and prisons; *k* = 13, OR = 3.86, 95% CI 2.67–3.97) had similar pooled odds to studies of current and former psychiatric patients (*k* = 58, OR = 3.23, 95% CI 2.64–3.96), but no estimates were provided specifically for general population samples. In contrast, the meta-analysis by Hubers et al. (2018) reported separate estimates by sample categories, including for general population samples (14 [17%] out of all 81 samples).

† After conversion, this effect size (Cohen's *d*) translates into OR = 2.48 (95% CI 1.29–4.67).

‡ The meta-analysis by Hill et al. (2020) considered exposure to suicide, suicide attempt, and suicidal behaviour (a composite measure of suicide and suicide attempt) by any relative, including parents. The authors found that, for exposure to suicide, the degree of relationship did not materially affect the strength of associations. The meta-analysis by Calderaro et al. (2022), which includes a larger pooled sample (*n* = 17.5 *vs*. 13.5 million), only examined exposure to parental death by suicide.

§ Conner et al. (2019) included 35 articles (*n* = 5594) that used the psychological autopsy approach to examine associations with mood and substance use disorders (anxiety, psychotic, and personality disorders were not considered). No study-level data were provided for any of the risk factors; only pooled effect sizes were reported. The meta-analysis by Moitra et al. (2021) included 20 studies (18 [90%] of which were psychological autopsy studies) covering 18,000 individuals and analysed associations for major depression, dysthymia, bipolar disorder, anxiety disorders, and schizophrenia (substance use and personality disorders were not considered). Using a multilevel meta-regression approach, the authors adjusted their estimates for a range of covariates (ie, sex, age, disorder type, study design, estimate adjustment, and use of psychological autopsy), which yields smaller pooled effect sizes compared to a standard meta-analytic approach. Too et al. (2019) synthesised 13 register-based studies (*n* = 6.5 million) and provided pooled estimates for mood, anxiety, psychotic, personality, and substance use disorders. We selected the review by Too et al. (2019) because it covers the broadest range of mental disorders, comprises the largest pooled sample, provides study-level data, and adopts a standard meta-analytic approach.

Overall, we gave preference to meta-analyses that examined multiple psychiatric disorders because this would facilitate comparisons of effect sizes between psychiatric disorders (examined using the same methods in one meta-analytic review) as opposed to comparing effects sizes for single disorders derived from different meta-analyses using contrasting methods and inclusion criteria (eg, psychotic disorders in one meta-analysis and substance use disorders in another).

Table S4. ROBIS scoring system.

Scoring	Each item per domain has five response options: yes (Y), probably yes (PY), no information (NI), probably no (PN), and no (N), with Y and PY indicating low concerns.
Domain	Bias within each domain was determined as:<br>- Low risk: if all items were scored Y or PY<br>- Unclear risk: if a single item was scores as PN, N or NI<br>- High risk: if two or more items were scored as PN, N or NI
Overall	Overall risk of bias regarding each meta-analysis was judged as:<br>- Low risk: if all four domains were judged as low risk or only one as unclear risk<br>- Moderate risk: if two or more domains were judged as unclear risk<br>- High risk: if one or more domains were judged as high risk

Note. Scoring system based on Melo et al. (2019).

Table S5. Overlapping meta-analyses that were excluded, by domain.

Sociodemographic risk factors (*k* = 8)

Amiri, S. (2022). Prevalence of suicide in immigrants/refugees: a systematic review and meta-analysis. *Archives of Suicide Research*, *26*(2), 370-405.

Duarte, D., El-Hagrassy, M.M., Couto, T.C.e., Gurgel, W., Fregni, F., & Correa, H. (2020). Male and female physician suicidality: a systematic review and meta-analysis. *JAMA Psychiatry, 77*(6), 587-597.

Dutheil, F., Aubert, C., Pereira, B., Dambrun, M., Moustafa, F., Mermillod, M., Baker, J.S., Trousselard, M., Lesage, F.X., & Navel, V. (2019). Suicide among physicians and health-care workers: a systematic review and meta-analysis. *PLoS ONE, 14*(12), e0226361.

Klingelschmidt, J., Milner, A., Khireddine-Medouni, I., Witt, K., Alexopoulos, E.C., Toivanen, S., LaMontagne, A.D., Chastang, J.F., & Niedhammer, I. (2018). Suicide among agricultural, forestry, and fishery workers: a systematic literature review and meta-analysis. *Scandinavian Journal of Work, Environment & Health, 44*(1), 3-15.

Milner, A., Page, A., & Lamontagne, A.D. (2013). Long-term unemployment and suicide: a systematic review and meta-analysis. *PLoS ONE, 8*(1), e51333.

Milner, A., Page, A., & LaMontagne, A.D. (2014). Cause and effect in studies on unemployment, mental health and suicide: a meta-analytic and conceptual review. *Psychological Medicine, 44*(5), 909-917.

Milner, A.J., Spittal, M.S., Pirkis, J., & LaMontagne, A.D. (2016). Does gender explain the relationship between occupation and suicide? Findings from a meta-analytic study. *Community Mental Health Journal, 52*(5), 568-573.

Wu, A., Wang, J.Y., & Jia, C.X. (2015). Religion and completed suicide: a meta-analysis. *PLoS ONE, 10*(6), e0131715.

Physical risk factors (*k* = 12)

Amiri, S., & Behnezhad, S. (2020). Cancer diagnosis and suicide mortality: a systematic review and meta-analysis. *Archives of Suicide Research*, *24*, S94-112.

Bell, G.S., Gaitatzis, A., Bell, C.L., Johnson, A.L., & Sander, J.W. (2009). Suicide in people with epilepsy: how great is the risk? *Epilepsia, 50*(8), 1933-1942.

Calati, R., Di Mattei, V., & Courtet, P. (2017). Risk of suicide mortality among cancer patients: a meta-analysis of observational studies. *European Psychiatry, 41*, S290-291.

Chi, C.C., Chen, T.H., Wang, S.H., & Tung, T.H. (2017). Risk of suicidality in people with psoriasis: a systematic review and meta-analysis of cohort studies. *American Journal of Clinical Dermatology, 18*(5), 621-627.

Elamoshy, R., Bird, Y., Thorpe, L., & Moraros, J. (2018). Risk of depression and suicidality among diabetic patients: a systematic review and meta-analysis. *Journal of Clinical Medicine*, *7*(11), 445.

Guo, Z., Gan, S., Li, Y., Gu, C., Xiang, S., Zhou, J., Gong, L., Chan, F.L., & Wang, S. (2018). Incidence and risk factors of suicide after a prostate cancer diagnosis: a meta-analysis of observational studies. *Prostate Cancer and Prostatic Diseases, 21*(4), 499-508.

Guo, Z., Gu, C., Li, S., Gan, S., Li, Y., Xiang, S., Gong, L., & Wang, S. (2021). Incidence and risk factors of suicide among patients diagnosed with bladder cancer: a systematic review and meta-analysis. *Urologic Oncology, 39*(3), 171-179.

Manouchehrinia, A., Tanasescu, R., Tench, C.R., & Constantinescu, C.S. (2016). Mortality in multiple sclerosis: meta-analysis of standardised mortality ratios. *Journal of Neurology, Neurosurgery and Psychiatry, 87*(3), 324-331.

Ravaioli, A., Crocetti, E., Mancini, S., Baldacchini, F., Giuliani, O., Vattiato, R., Bucchi, L., & Falcini, F. (2020). Suicide death among cancer patients: new data from northern Italy, systematic review of the last 22 years and meta-analysis. *European Journal of Cancer*, *125*, 104-113.

Singh, S., Taylor, C., Kornmehl, H., & Armstrong, A.W. (2017). Psoriasis and suicidality: a systematic review and meta-analysis. *Journal of the American Academy of Dermatology, 77*(3), 425-440.

Wang, Y., Tang, S., Xu, S., Weng, S., & Liu, Z. (2016). Association between diabetes and risk of suicide death: a meta-analysis of 3 million participants. *Comprehensive Psychiatry, 71*, 11-16.

Zhang, C., Byrne, G., Lee, T., Singer, J., Giustini, D., & Bressler, B. (2018). Incidence of suicide in inflammatory bowel disease: a systematic review and meta-analysis. *Journal of the Canadian Association of Gastroenterology, 1*(3), 107-114.

Psychiatric risk factors (*k* = 18)

Amiri, S., & Behnezhad, S. (2020). Alcohol use and risk of suicide: a systematic review and meta-analysis. *Journal of Addictive Diseases, 38*(2), 200–213.

Arsenault-Lapierre, G., Kim, C., & Turecki, G. (2004). Psychiatric diagnoses in 3275 suicides: a meta-analysis. *BMC Psychiatry, 4*, 37.

Brown, S. (1997). Excess mortality of schizophrenia: a meta-analysis. *British Journal of Psychiatry, 171*(6), 502-508.

Conner, K.R., Bridge, J.A., Davidson, D.J., Pilcher, C., & Brent, D.A. (2019). Metaanalysis of mood and substance use disorders in proximal risk for suicide deaths. *Suicide and Life-Threatening Behavior*, *49*(1), 278-292.

Correll, C.U., Solmi, M., Croatto, G., Schneider, L.K., Rohani-Montez, S.C., Fairley, L., Smith, N., Bitter, I., Gorwood, P., Taipale, H., & Tiihonen, J. (2022). Mortality in people with schizophrenia: a systematic review and meta-analysis of relative risk and aggravating or attenuating factors. *World Psychiatry, 21*(2), 248-271.

Darvishi, N., Farhadi, M., Haghtalab, T., & Poorolajal, J. (2015). Alcohol-related risk of suicidal ideation, suicide attempt, and completed suicide: a meta-analysis. *PLoS ONE, 10*(5), e0126870.

Harris, E.C., & Barraclough, B. (1997). Suicide as an outcome for mental disorders: a meta-analysis. *British Journal of Psychiatry, 170*(3), 205-228.

Hayes, J.F., Miles, J., Walters, K., King, M., & Osborn, D.P.J. (2015). A systematic review and meta-analysis of premature mortality in bipolar affective disorder. *Acta Psychiatrica Scandinavica, 131*(6), 417-425.

Kanwar, A., Malik, S., Prokop, L.J., Sim, L.A., Feldstein, D., Wang, Z., & Murad, M.H. (2013). The association between anxiety disorders and suicidal behaviors: a systematic review and meta-analysis. *Depression and Anxiety, 30*(10), 917-929.

Keshaviah, A., Edkins, K., Hastings, E.R., Krishna, M., Franko, D.L., Herzog, D.B., Thomas, J.J., Murray, H.B., & Eddy, K.T. (2014). Re-examining premature mortality in anorexia nervosa: a meta-analysis redux. *Comprehensive Psychiatry*, *55*(8), 1773-1784.

Moitra, M., Santomauro, D., Degenhardt, L., Collins, P.Y., Whiteford, H., Vos, T., & Ferrari, A. (2021). Estimating the risk of suicide associated with mental disorders: a systematic review and meta-regression analysis. *Journal of Psychiatric Research, 137*, 242-249.

Neeleman, J. (2001). A continuum of premature death. Meta-analysis of competing mortality in the psychosocially vulnerable. *International Journal of Epidemiology, 30*(1), 154-162.

Panagioti, M., Gooding, P.A., & Tarrier, N. (2012). A meta-analysis of the association between posttraumatic stress disorder and suicidality: the role of comorbid depression. *Comprehensive Psychiatry, 53*(7), 915-930.

Pompili, M., Girardi, P., Ruberto, A., & Tatarelli, R. (2005). Suicide in borderline personality disorder: a meta-analysis. *Nordic Journal of Psychiatry, 59*(5), 319–324.

Poorolajal, J., Haghtalab, T., Farhadi, M., & Darvishi, N. (2016). Substance use disorder and risk of suicidal ideation, suicide attempt and suicide death: a meta-analysis. *Journal of Public Health, 38*(3), 282-291.

Preti, A., Rocchi, M.B.L., Sisti, D., Camboni, M.V., & Miotto, P. (2011). A comprehensive meta-analysis of the risk of suicide in eating disorders. *Acta Psychiatrica Scandinavica, 124*(1), 6-17.

Saha, S., Chant, D., & McGrath, J. (2007). A systematic review of mortality in schizophrenia: is the differential mortality gap worsening over time? *Archives of General Psychiatry, 64*(10), 1123-1131.

Wilcox, H.C., Conner, K.R., & Caine, E.D. (2004). Association of alcohol and drug use disorders and completed suicide: an empirical review of cohort studies. *Drug and Alcohol Dependence, 76*(7), S11-19.

Suicide-related risk factors (*k* = 3)

Geulayov, G., Gunnell, D., Holmen, T.L., & Metcalfe, C. (2012). The association of parental fatal and non-fatal suicidal behaviour with offspring suicidal behaviour and depression: a systematic review and meta-analysis. *Psychological Medicine, 42*(8), 1567-1580.

Hill, N.T.M., Robinson, J., Pirkis, J., Andriessen, K., Krysinska, K., Payne, A., Boland, A., Clarke, A., Milner, A., Witt, K., Krohn, S., & Lampit, A. (2020). Association of suicidal behavior with exposure to suicide and suicide attempt: a systematic review and multilevel meta-analysis. *PLoS Medicine*, *17*(3), e1003074.

McHugh, C., Corderoy, A., Ryan, C., Hickie, I., & Large, M. (2019). Association between suicidal ideation and suicide: meta-analyses of odds ratios, sensitivity, specificity and positive predictive value. *BJPsych Open*, *5*(2), e18.

Other risk factors (*k* = 5)

Amiri, S., & Behnezhad, S. (2018). Body mass index and risk of suicide: a systematic review and meta-analysis. *Journal of Affective Disorders*, *238*, 615-625.

Li, D.J., Yang, X.L., Ge, Z., Hao, Y.C., Wang, Q.Q., Liu, F.C., Gu, D.F., & Huang, J.F. (2012). Cigarette smoking and risk of completed suicide: a meta-analysis of prospective cohort studies. *Journal of Psychiatric Research, 46*(10), 1257-1266.

Liu, R.T., Steele, S.J., Hamilton, J.L., Do, Q.B.P., Furbish, K., Burke, T.A., Martinez, A.P., & Gerlus, N. (2020). Sleep and suicide: a systematic review and meta-analysis of longitudinal studies. *Clinical Psychology Review*, *81*, 101895.

Pigeon, W.R., Pinquart, M., & Conner, K. (2012). Meta-analysis of sleep disturbance and suicidal thoughts and behaviors. *Journal of Clinical Psychiatry, 73*(9), 1160-1167.

Poorolajal, J., & Darvishi, N. (2016). Smoking and suicide: a meta-analysis. *PLoS ONE, 11*(7), e0156348.

Table S6. Characteristics of included meta-analyses.

Meta-analysis	Risk factor	*k*	*n*	Date range	Designs	ES	Ages	By sex	ROBIS	Outcome
Alvarez Munoz 2020	Dementia	16	NR	2000–2018	All	OR	Adults	No	M	I, A, S
Amiri 2022	Unemployment	21	NR	1987–2019	All	OR	Adults	No	H	I, A, S
Anglemyer 2014	Access to firearms	14	NR	1988–2005	All	OR	All	Yes	L	S, M
Batty 2022	State care in childhood	4	534,890	1995–2018	Cohort[*]	RR	Adults	Yes	L	S, M
Calderaro 2021	Parental death by suicide	10	17,483,726	2002–2018	All	RR	All	No	L	A, S
Dong 2021	Sleep disturbances	5	1,088,983	2005–2017	Cohort	RR	All	No	L	A, S
Du 2020	Parkinson's disease	4	NR	1994–2017	All	SMR	Adults	No	H	S
Echeverria 2021[§]	Smoking	15	2,407,677	1973–2017	Cohort[*]	RR	All	Yes	M	I, A, S
Ferrari 2014	Anorexia nervosa	9	NR	1989–2009	All	RR	All	Yes	M	S
Fralick 2019	Concussion	6	14,363,256	2001–2016	All	RR	All	No	L	I, A, S
Heinrich 2022	Cancer	28	22,407,690	1979–2021	Cohort	SMR	All	Yes	L	S
Hubers 2018	Suicidal ideation	14	145,411	1996–2016	All	RR	Adults	No	L	S
Hung 2023	Psoriasis	7	7,622,906	2010–2019	Cohort	HR	All	No	M	I, A, S
Kyung-Sook 2018[**]	Marital status	36	117,387,778	2001–2015	All	OR	Adults	Yes	L	S
Li 2011	Socioeconomic status[††]	6	NR	1994–2007	All	RR	All	Yes	H	S
Milner 2013	Occupation	34	NR	1979–2012	All	IRR	Adults	Yes	L	S
Milner 2018	Job stressors	6	62,213	2007–2014	All	OR	Adults	No	L	I, A, S
Nevalainen 2016[§]	Epilepsy	6	NR	1997–2013	Cohort	RR	All	No	L	S, M
Perera 2016	Body mass index	15	10,130,420	2006–2014	Cohort	HR	Adults	No	L	I, A, S
Phan 2020	Hidradenitis suppurativa	4	114,626,714	2017–2018	All	OR	All	No	H	S, P
Poorolajal 2022	Religious affiliation	14	7,542,560	2000–2020	All	RR	All	No	H	I, A, S
Richardson 2013[§]	Unsecured financial debt	4	1069	2006–2010	CC[†]	OR	Adults	No	H	S, P
Sampaio 2019	COPD	5	376,879	2002–2015	CC	OR	All	No	M	S
Septier 2019	ADHD	4	398,359	2013–2017	All	OR	All	No	L	I, A, S
Shen 2019	Multiple sclerosis	16	766,456	1992–2018	All	IRR	All	Yes	M	S
Skinner 2020	Criminal offending	15	602,347	1998–2017	All	OR	All	No	M	S

Too 2019	Mental disorders[††]	13	6,475,685	2000–2017	All[‡]	RR	All	No	L	S
Troya 2022	Ethnic minority	51	1,028,561	2000–2020	All	IRR	All	Yes	L	S
Vyas 2021	Stroke	14	18,090,596	2001–2020	All	RR	Adults	No	M	A, S
Wang 2017	Diabetes	28	28,057,822	1991–2017	Cohort	RR	All	Yes	L	S
Xiong 2022	Inflammatory bowel disease	17	7,768,423	1992–2021	All	RR	All	Yes	L	A, S
Yoshimasu 2008	Suicide attempt/self-harm	11	3353	1999–2006	CC[†]	OR	All	No	H	S
Zhang 2019	Asthma	8	18,277,066	2010–2017	All	OR	All	No	M	I, A, S

Note. Several meta-analyses reported associations for multiple outcomes; data presented refer to studies on suicide mortality only. ES = effect size statistic (OR = odds ratio; RR = relative risk or risk ratio; SMR = standardised mortality ratio; HR = hazard ratio; IRR = incidence rate ratio); ROBIS = Risk of Bias in Systematic Reviews (L = low risk of bias, M = moderate risk of bias, H = high risk of bias); CC = case-control studies; I = suicidal ideation; A = suicide attempt; S = suicide mortality; M = all-cause mortality or mortality from causes other than suicide; P = psychiatric disorders; COPD = chronic obstructive pulmonary disease; ADHD = attention-deficit hyperactivity disorder; NR = not reported.

[*] Prospective cohort studies only.

[†] Psychological autopsy studies only.

[‡] Register-based studies only.

[§] Additional data provided by study authors.

[**] No study-level data available.

[††] Multiple risk factors from this meta-analysis.

Table S7. Included primary studies by country income level.

Meta-analysis	Risk factor	Studies		Countries	
		Total	Of which from HICs	Total	Of which HICs
Alvarez Munoz 2020	Dementia	16	15 (94%)	11	10 (91%)[a]
Amiri 2022	Unemployment	21	19 (90%)	14	13 (93%)[b]
Anglemyer 2014	Access to firearms	14	14 (100%)	3	3 (100%)
Batty 2022	State care in childhood	4	4 (100%)	3	3 (100%)
Calderaro 2021	Parental death by suicide	10	10 (100%)	5	5 (100%)
Dong 2021	Sleep disturbances	5	5 (100%)	5	5 (100%)
Du 2020	Parkinson’s disease	4	3 (75%)	4	3 (75%)[c]
Echeverria 2021	Smoking	15	15 (100%)	7	7 (100%)
Ferrari 2014	Anorexia nervosa	9	9 (100%)	5	5 (100%)
Fralick 2019	Concussion	6	6 (100%)	4	4 (100%)
Heinrich 2022	Cancer	28	28 (100%)	16	16 (100%)
Hubers 2018	Suicidal ideation	14	12 (86%)	10	8 (80%)[d]
Hung 2023	Psoriasis	7	7 (100%)	4	4 (100%)
Kyung-Sook 2018	Marital status	36	27 (75%)	25	20 (80%)[e]
Li 2011	Socioeconomic status	6	6 (100%)	5	5 (100%)
Milner 2013	Occupation	34	34 (100%)	13	13 (100%)
Milner 2018	Job stressors	6	6 (100%)	5	5 (100%)
Nevalainen 2016	Epilepsy	6	6 (100%)	5	5 (100%)
Perera 2016	BMI (underweight)	9	9 (100%)	5	5 (100%)
Phan 2020	Hidradenitis suppurativa	4	4 (100%)	3	3 (100%)
Poorolajal 2022	Religious affiliation	14	11 (79%)	8	6 (75%)[f]
Richardson 2013	Unsecured financial debt	4	4 (100%)	1	1 (100%)
Sampaio 2019	COPD	5	5 (100%)	3	3 (100%)
Septier 2019	ADHD	4	4 (100%)	4	4 (100%)
Shen 2019	Multiple sclerosis	16	16 (100%)	9	9 (100%)
Skinner 2020	Criminal offending	15	14 (93%)	8	7 (88%)[g]
Too 2019	Mental disorders	13	13 (100%)	6	6 (100%)
Troya 2022	Ethnic minority	51	44 (86%)	11	7 (64%)[h]
Vyas 2021	Stroke	14	13 (93%)	9	8 (89%)[i]
Wang 2017	Diabetes	28	28 (100%)	17	17 (100%)
Xiong 2022	Inflammatory bowel disease	17	17 (100%)	21	21 (100%)
Yoshimasu 2008	Suicide attempt/self-harm	11	8 (73%)	8	6 (75%)[j]
Zhang 2019	Asthma	8	6 (75%)	5	4 (80%)[k]

Note. Numbers refer to primary studies included in the meta-analyses on suicide mortality only. HICs, high-income countries. Low-income and middle-income countries were: [a] Sri Lanka; [b] India; [c] Serbia; [d] Colombia and the Philippines; [e] China, India, Iran, Pakistan, and Serbia; [f] Indonesia and China; [g] Russia; [h] India, Brazil, Russia, and Fiji; [i] China. [j] India and China; [k] China.

Table S8. Definition of exposures.

Meta-analysis	Risk factor	Definition of exposure
Alvarez Munoz 2020	Dementia	Diagnosis of dementia
Amiri 2022	Unemployment	Not employed
Anglemyer 2014	Access to firearms	Firearm ownership or accessibility
Batty 2022	State care in childhood	Temporary out-of-home care in childhood (excluding adoption)
Calderaro 2021	Parental death by suicide	History of parental death by suicide
Dong 2021	Sleep disturbances	Insomnia and sleep apnea
Du 2020	Parkinson's disease	Diagnosis of Parkinson's disease
Echeverria 2021	Smoking	Current tobacco smoking
Ferrari 2014	Anorexia nervosa	Diagnosis of anorexia nervosa
Fralick 2019	Concussion	Diagnosis of concussion (including mild traumatic brain injury)
Heinrich 2022	Cancer	Diagnosis of cancer
Hubers 2018	Suicidal ideation	Suicidal ideation (suicidal thoughts, ranging from death wish to suicide plans)
Hung 2023	Psoriasis	Diagnosis of psoriasis
Kyung-Sook 2018	Marital status	Not married, including being widowed, divorced, separated, and single
Li 2011	Low income	Lowest income level
	Low education level	Less than secondary schooling
Milner 2013	Occupation	Lowest skill level occupation (ISCO group 9; occupations that require simple and routine physical or manual tasks)
Milner 2018	Job stressors	Poor colleague or supervisor support, low job control, high psychological job demands, job insecurity, and job strain
Nevalainen 2016	Epilepsy	Diagnosis of epilepsy
Perera 2016	BMI (underweight)	BMI less than 18.5 kg/m^2
Phan 2020	Hidradenitis suppurativa	Diagnosis of hidradenitis suppurativa
Poorolajal 2022	No religious affiliation	Religious beliefs or practices (inversed)
Richardson 2013	Unsecured financial debt	Unmanageable financial debt
Sampaio 2019	COPD	Diagnosis of COPD
Septier 2019	ADHD	Diagnosis of ADHD
Shen 2019	Multiple sclerosis	Diagnosis of multiple sclerosis
Skinner 2020	Criminal offending	Current or previous contact with the criminal justice system (excluding those currently incarcerated)
Too 2019	Mood disorders	Diagnosis of mood disorders (including depression and bipolar disorder)
	Anxiety disorders	Diagnosis of any anxiety disorder (including obsessive compulsive disorder)

	Psychotic disorders	Diagnosis of schizophrenia spectrum disorder
	Substance use disorders	Diagnosis of alcohol use disorder and drug use disorders
	Personality disorders	Diagnosis of any personality disorder
Troya 2022	Ethnic minority	Ethnic minority groups, including migrant groups and indigenous peoples
Vyas 2021	Stroke	Diagnosis of stroke
Wang 2017	Diabetes	Diagnosis of diabetes (type 1 and type 2)
Xiong 2022	Inflammatory bowel disease	Diagnosis of inflammatory bowel disease (ulcerative colitis and Crohn's disease)
Yoshimasu 2008	Suicide attempt/self-harm	Previous suicide attempts and deliberate self-harm
Zhang 2019	Asthma	Diagnosis of asthma

Table S9. Risk factors for suicide stratified by sex.

Risk factor	Statistic	Effect size (95% CI)	
		Males	Females
Cancer	SMR	1.74 (1.52–2.00)	1.59 (1.27–1.97)
Diabetes	RR	1.39 (1.23–1.57)	1.36 (1.10–1.69)
Inflammatory bowel disease	RR	1.01 (0.81–1.27)	1.44 (1.17–1.78)
Multiple sclerosis	IRR	1.54 (1.19–1.99)	1.74 (1.51–1.99)
Anorexia nervosa	RR	6.20 (2.80–11.80)	7.70 (3.70–15.90)
Ethnic minority	IRR	1.20 (0.80–2.00)	1.40 (0.80–2.40)
Marital status	OR	2.47 (2.42–2.53)	1.74 (1.68–1.80)
Low skill level occupation	IRR	1.30 (0.94–1.80)	1.14 (0.82–1.60)
Low income	RR	2.18 (1.47–3.22)	1.45 (0.95–2.21)
Low education level	RR	2.42 (1.03–5.70)	1.48 (0.94–2.34)
Access to firearms	OR	3.71 (1.61–9.00)	3.56 (0.53–21.12)
Smoking	RR	2.06 (1.62–2.62)	2.51 (2.06–3.04)
State care in childhood	RR	3.37 (1.79–6.37)	5.34 (3.64–7.82)

Table S10. Meta-analyses that additionally examined associations with outcomes other than suicide mortality.

Meta-analysis	Risk factor	ES	Suicide mortality	Suicide attempt	Suicidal ideation	Non-suicide mortality	Psychiatric disorder
Alvarez Munoz 2020	Dementia	OR	1.3 (0.8–2.1)	2.2 (1.0–5.0)	1.4 (0.8–2.4)		
Amiri 2022	Unemployment	OR	1.9 (1.4–2.5)	1.5 (1.3–1.9)	1.9 (1.6–2.3)		
Anglemyer 2014	Access to firearms	OR	3.2 (2.4–4.4)			2.0 (1.6–3.0)	
Batty 2022	State care in childhood	RR	3.4 (2.4–4.7)			2.2 (1.6–3.0)	
Calderaro 2021	Parental death by suicide	RR	3.0 (2.5–3.5)	1.8 (1.6–2.0)			
Dong 2021	Sleep disturbances	RR	1.8 (1.3–2.4)	3.5 (3.1–4.1)			
Echeverria 2021	Smoking	RR	2.4 (2.1–2.8)	1.7 (0.7–4.0)	1.8 (1.2–2.8)		
Hung 2023	Psoriasis	HR	1.3 (0.9–2.0)	1.2 (0.9–1.6)	1.7 (0.9–3.1)		
Milner 2018	Job stressors	OR	1.2 (1.0–1.3)	1.2 (1.1–1.2)	1.2 (1.1–1.2)		
Nevalainen 2016	Epilepsy	RR	2.9 (2.2–3.8)			2.4 (1.9–3.0)	
Phan 2020	Hidradenitis suppurativa	OR	2.1 (1.3–3.4)				1.8 (1.6–1.9)
Poorolajal 2022	No religious affiliation	RR	2.4 (1.9–2.9)	1.2 (1.1–1.3)	1.2 (1.1–1.3)		
Richardson 2013	Unsecured financial debt	OR	7.9 (5.2–12.0)				3.2 (2.9– 3.6)
Septier 2019	ADHD	OR	6.7 (3.2–13.8)	2.4 (1.6–3.4)	3.5 (2.9–4.3)		
Vyas 2021	Stroke	RR	1.6 (1.4–1.8)	2.1 (1.7–2.6)			
Xiong 2022	Inflammatory bowel disease	RR	1.3 (1.1–1.4)	1.4 (1.1–1.8)			
Zhang 2019	Asthma	OR	1.3 (1.1–1.6)	1.6 (1.3–1.9)	1.5 (1.4–1.7)		

Note. ES = effect size statistic (OR = odds ratio; RR = relative risk or risk ratio; HR = hazard ratio). Non-suicide mortality includes both all-cause mortality (state care in childhood and epilepsy) and mortality from causes other than suicide (homicide for access to firearms). Fralick (2019) and Perera (2016) examined associations with suicidal ideation and suicide attempt but did not meta-analyse results for these outcomes.

Figure S1. Association between income and suicide, men and women combined (Li et al., 2011).

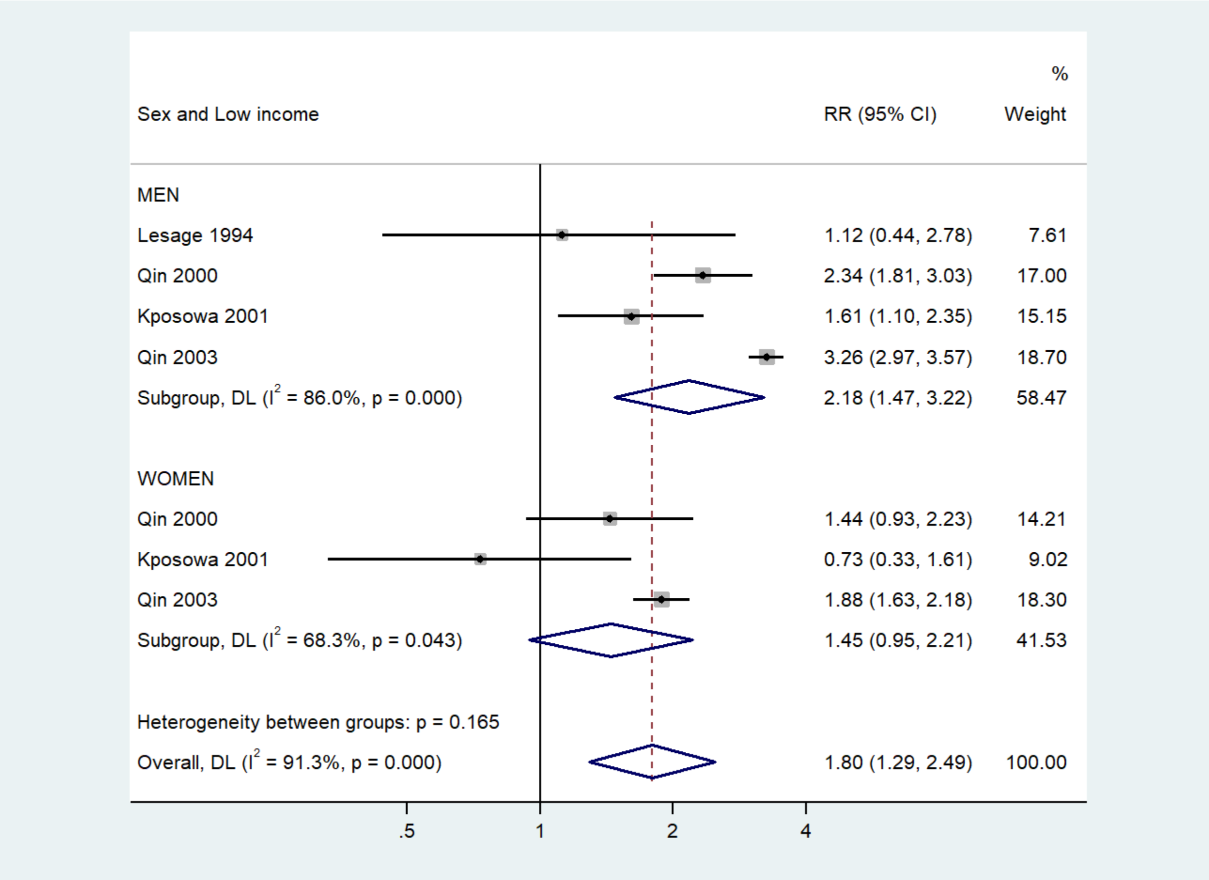

RR = relative risk; CI = confidence interval.

Figure S2. Association between education and suicide, men and women combined (Li et al., 2011).

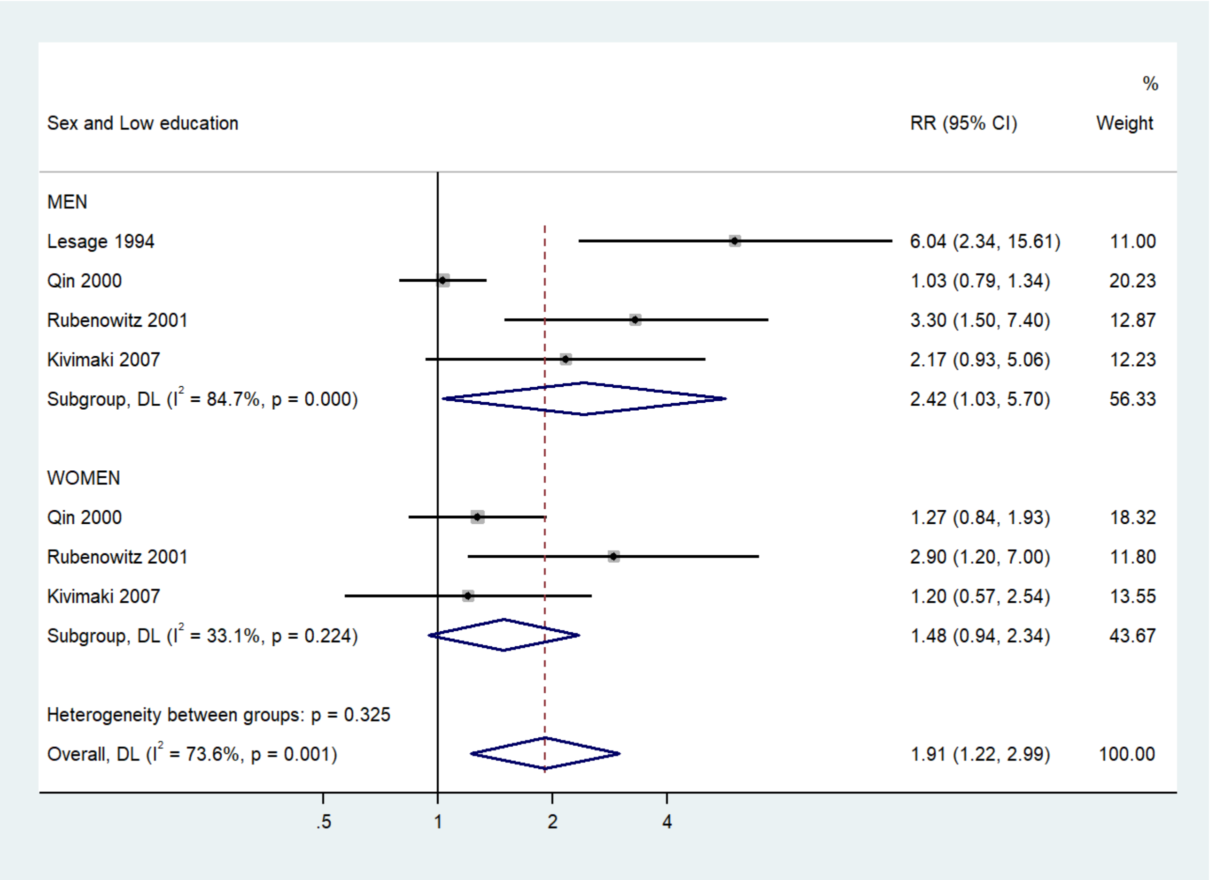

RR = relative risk; CI = confidence interval.

Figure S3. Study selection.

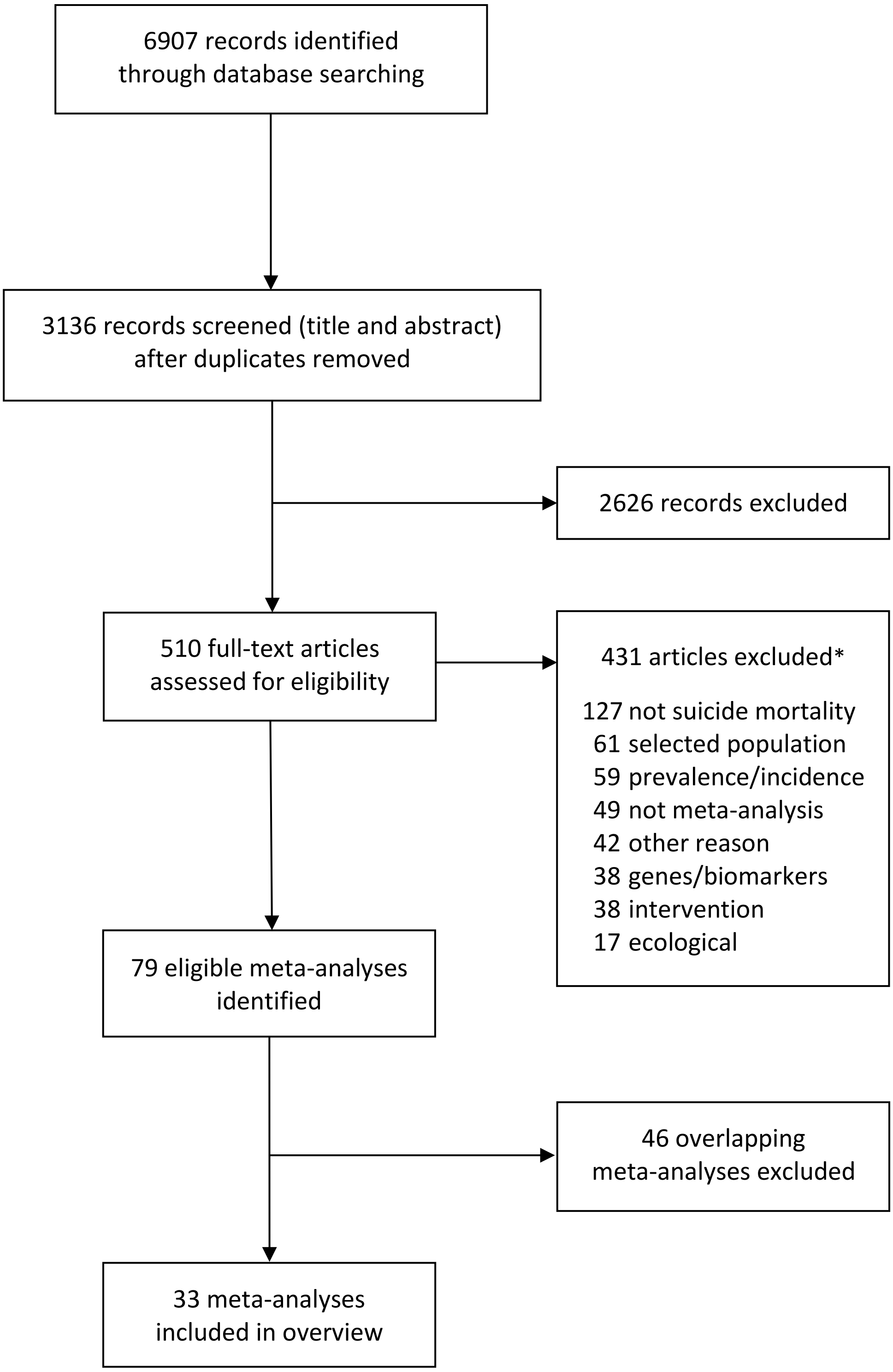

* Studies can be excluded for multiple reasons; numbers listed are based on the major reasons for exclusion.

Supplementary references

Amiri, S. (2022). Prevalence of suicide in immigrants/refugees: a systematic review and meta-analysis. *Archives of Suicide Research*, *26*(2), 370-405.

Amiri, S., & Behnezhad, S. (2018). Body mass index and risk of suicide: a systematic review and meta-analysis. *Journal of Affective Disorders*, *238*, 615-625.

Amiri, S., & Behnezhad, S. (2020). Cancer diagnosis and suicide mortality: a systematic review and meta-analysis. *Archives of Suicide Research*, *24*, S94-112.

Bentley, K.H., Franklin, J.C., Ribeiro, J.D., Kleiman, E.M., Fox, K.R., & Nock, M.K. (2016). Anxiety and its disorders as risk factors for suicidal thoughts and behaviors: a meta-analytic review. *Clinical Psychology Review*, *43*, 30-46.

Calderaro, M., Baethge, C., Bermpohl, F., Gutwinski, S., Schouler-Ocak, M., & Henssler, J. (2022). Offspring's risk for suicidal behaviour in relation to parental death by suicide: systematic review and meta-analysis and a model for familial transmission of suicide. *British Journal of Psychiatry*, *220*(3), 121-129.

Chang, B.P., Franklin, J.C., Ribeiro, J.D., Fox, K.R., Bentley, K.H., Kleiman, E.M., & Nock, M.K. (2016). Biological risk factors for suicidal behaviors: a meta-analysis. *Translational Psychiatry*, *6*(9), e887.

Conner, K.R., Bridge, J.A., Davidson, D.J., Pilcher, C., & Brent, D.A. (2019). Metaanalysis of mood and substance use disorders in proximal risk for suicide deaths. *Suicide and Life-Threatening Behavior*, *49*(1), 278-292.

Dong, M., Li, L., Sha, S., Zhang, L., Zhang, Q.G., Ungvari, G.S., Balbuena, L., & Xiang, Y.T. (2021). Sleep disturbances and the risk of incident suicidality: a systematic review and meta-analysis of cohort studies. *Psychosomatic Medicine*, *83*(7), 739-745.

Elamoshy, R., Bird, Y., Thorpe, L., & Moraros, J. (2018). Risk of depression and suicidality among diabetic patients: a systematic review and meta-analysis. *Journal of Clinical Medicine*, *7*(11), 445.

Ferrari, A.J., Norman, R.E., Freedman, G., Baxter, A.J., Pirkis, J.E., Harris, M.G., Page, A., Carnahan, E., Degenhardt, L., Vos, T., & Whiteford, H.A. (2014). The burden attributable to mental and substance use disorders as risk factors for suicide: findings from the Global Burden of Disease Study 2010. *PLoS ONE*, *9*(4), e91936.

Franklin, J.C., Ribeiro, J.D., Fox, K.R., Bentley, K.H., Kleiman, E.M., Huang, X., Musacchio, K.M., Jaroszewski, A.C., Chang, B.P., & Nock, M.K. (2017). Risk factors for suicidal thoughts and behaviors: a meta-analysis of 50 years of research. *Psychological Bulletin*, *143*(2), 187-232.

Glenn, C.R., Kleiman, E.M., Cha, C.B., Deming, C.A., Franklin, J.C., & Nock, M.K. (2018). Understanding suicide risk within the Research Domain Criteria (RDoC) framework: a meta-analytic review. *Depression and Anxiety*, *35*(1), 65-88.

Harris, L.M., Broshek, C.E., & Ribeiro, J.D. (2022). Does body mass index confer risk for future suicidal thoughts and behaviors? A meta-analysis of longitudinal studies. *Current Obesity Reports*, *11*(2), 45-54.

Harris, L.M., Huang, X.Y.N., Linthicum, K.P., Bryen, C.P., & Ribeiro, J.D. (2020). Sleep disturbances as risk factors for suicidal thoughts and behaviours: a meta-analysis of longitudinal studies. *Scientific Reports*, *10*(1), 13888

Heinrich, M., Hofmann, L., Baurecht, H., Kreuzer, P.M., Knuttel, H., Leitzmann, M.F., & Seliger, C. (2022). Suicide risk and mortality among patients with cancer. *Nature Medicine*, *28*(4), 852-859.

Hill, N.T.M., Robinson, J., Pirkis, J., Andriessen, K., Krysinska, K., Payne, A., Boland, A., Clarke, A., Milner, A., Witt, K., Krohn, S., & Lampit, A. (2020). Association of suicidal behavior with exposure to suicide and suicide attempt: a systematic review and multilevel meta-analysis. *PLoS Medicine*, *17*(3), e1003074.

Huang, X., Fox, K.R., Ribeiro, J.D., & Franklin, J.C. (2018). Psychosis as a risk factor for suicidal thoughts and behaviors: a meta-analysis of longitudinal studies. *Psychological Medicine*, *48*(5), 765-776.

Huang, X.Y.N., Ribeiro, J.D., Musacchio, K.M., & Franklin, J.C. (2017). Demographics as predictors of suicidal thoughts and behaviors: a meta-analysis. *PLoS ONE*, *12*(7), e0180793.

Huang, X.Y.N., Rootes-Murdy, K., Bastidas, D.M., Nee, D.E., & Franklin, J.C. (2020). Brain differences associated with self-injurious thoughts and behaviors: a meta-analysis of neuroimaging studies. *Scientific Reports*, *10*(1), 2404.

Hubers, A.A., Moaddine, S., Peersmann, S.H., Stijnen, T., van Duijn, E., van der Mast, R.C., Dekkers, O.M., & Giltay, E.J. (2018). Suicidal ideation and subsequent completed suicide in both psychiatric and non-psychiatric populations: a meta-analysis. *Epidemiol Psychiatr Sci*, *27*(2), 186-198.

Keshaviah, A., Edkins, K., Hastings, E.R., Krishna, M., Franko, D.L., Herzog, D.B., Thomas, J.J., Murray, H.B., & Eddy, K.T. (2014). Re-examining premature mortality in anorexia nervosa: a meta-analysis redux. *Comprehensive Psychiatry*, *55*(8), 1773-1784.

Li, Z.Y., Page, A., Martin, G., & Taylor, R. (2011). Attributable risk of psychiatric and socio-economic factors for suicide from individual-level, population-based studies: a systematic review. *Social Science & Medicine*, *72*(4), 608-616.

Liu, R.T., Steele, S.J., Hamilton, J.L., Do, Q.B.P., Furbish, K., Burke, T.A., Martinez, A.P., & Gerlus, N. (2020). Sleep and suicide: a systematic review and meta-analysis of longitudinal studies. *Clinical Psychology Review*, *81*, 101895.

McHugh, C., Corderoy, A., Ryan, C., Hickie, I., & Large, M. (2019). Association between suicidal ideation and suicide: meta-analyses of odds ratios, sensitivity, specificity and positive predictive value. *BJPsych Open*, *5*(2), e18.

Melo, G., Duarte, J., Pauletto, P., Porporatti, A.L., Stuginski-Barbosa, J., Winocur, E., Flores-Mir, C., & Canto, G.D. (2019). Bruxism: an umbrella review of systematic reviews. *Journal of Oral Rehabilitation*, *46*(7), 666-690.

Moitra, M., Santomauro, D., Degenhardt, L., Collins, P.Y., Whiteford, H., Vos, T., & Ferrari, A. (2021). Estimating the risk of suicide associated with mental disorders: a systematic review and meta-regression analysis. *Journal of Psychiatric Research*, *137*, 242-249.

Perera, S., Eisen, R.B., Dennis, B.B., Bawor, M., Bhatt, M., Bhatnagar, N., Thabane, L., de Souza, R., & Samaan, Z. (2016). Body mass index is an important predictor for suicide: results from a systematic review and meta-analysis. *Suicide and Life-Threatening Behavior*, *46*(6), 697-736.

Ravaioli, A., Crocetti, E., Mancini, S., Baldacchini, F., Giuliani, O., Vattiato, R., Bucchi, L., & Falcini, F. (2020). Suicide death among cancer patients: new data from northern Italy, systematic review of the last 22 years and meta-analysis. *European Journal of Cancer*, *125*, 104-113.

Ribeiro, J.D., Franklin, J.C., Fox, K.R., Bentley, K.H., Kleiman, E.M., Chang, B.P., & Nock, M.K. (2016). Self-injurious thoughts and behaviors as risk factors for future suicide ideation, attempts, and death: a meta-analysis of longitudinal studies. *Psychological Medicine*, *46*(2), 225-236.

Ribeiro, J.D., Huang, X.Y., Fox, K.R., & Franklin, J.C. (2018). Depression and hopelessness as risk factors for suicide ideation, attempts and death: meta-analysis of longitudinal studies. *British Journal of Psychiatry*, *212*(5), 279-286.

Smith, A.R., Velkoff, E.A., Ribeiro, J.D., & Franklin, J. (2019). Are eating disorders and related symptoms risk factors for suicidal thoughts and behaviors? A meta-analysis. *Suicide and Life-Threatening Behavior*, *49*(1), 221-239.

Too, L.S., Spittal, M.J., Bugeja, L., Reifels, L., Butterworth, P., & Pirkis, J. (2019). The association between mental disorders and suicide: a systematic review and meta-analysis of record linkage studies. *Journal of Affective Disorders*, *259*, 302-313.

Troya, M.I., Spittal, M.J., Pendrous, R., Crowley, G., Gorton, H.C., Russell, K., Byrne, S., Musgrove, R., Hannah-Swain, S., Kapur, N., & Knipe, D. (2022). Suicide rates amongst individuals from ethnic minority backgrounds: a systematic review and meta-analysis. *EClinicalMedicine*, *47*, 101399.

Wang, B., An, X.F., Shi, X.H., & Zhang, J.A. (2017). Suicide risk in patients with diabetes: a systematic review and meta-analysis. *European Journal of Endocrinology*, *177*(4), 169-181.

Wang, Y., Tang, S.M., Xu, S.S., Weng, S.H., & Liu, Z.C. (2016). Association between diabetes and risk of suicide death: a meta-analysis of 3 million participants. *Comprehensive Psychiatry*, *71*, 11-16.

Witte, T.K., Gauthier, J.M., Huang, X.Y.N., Ribeiro, J.D., & Franklin, J.C. (2018). Is externalizing psychopathology a robust risk factor for suicidal thoughts and behaviors? A meta-analysis of longitudinal studies. *Journal of Clinical Psychology*, *74*(9), 1607-1625.
